# Six-component synthesis and biological activity of novel spiropyridoindolepyrrolidine derivatives: A combined experimental and theoretical investigation

**DOI:** 10.3389/fchem.2022.949205

**Published:** 2022-09-21

**Authors:** Zinatossadat Hossaini, Marziyeh Mohammadi, Fatemeh Sheikholeslami-Farahani

**Affiliations:** ^1^ Department of Chemistry, Qaemshahr Branch, Islamic Azad University, Qaemshahr, Iran; ^2^ Department of Chemistry, Faculty of Science, Vali-e-Asr University of Rafsanjan, Rafsanjan, Iran; ^3^ Department of Chemistry, Firoozkooh Branch, Islamic Azad University, Firoozkooh, Iran

**Keywords:** Ag/Fe 3 O 4 /CdO@MWCNTs, vinilidene Meldrum’s acid, acetyl chloride spiropyridoindolepyrrolidine, six component reactions, isatins

## Abstract

*Petasites hybridus* rhizome water extract was used as green media for the preparation of Ag/Fe_3_O_4_/CdO@multi-walled carbon nanotubes magnetic nanocomposites (Ag/Fe_3_O_4_/CdO@MWCNTs MNCs), and its activity was evaluated by using in the one-pot multicomponent reaction of isatins, acetyl chloride, secondary amines, vinilidene Meldrum’s acid, primary amines, and malononitrile in an aqueous medium at room temperature for the generation of spiropyridoindolepyrrolidine as new derivatives with tremendous output. In addition, organic pollutant reduction of 4-nitrophenol (4-NP) was carried out by generated Ag/Fe_3_O_4_/CdO@MWCNTs in water at room temperature. The results displayed that Ag/Fe_3_O_4_/CdO@MWCNTs were reduced as pollutants of organic compounds in a short time. The synthesized spiropyridoindolepyrrolidine has an NH_2_ functional group that has acidic hydrogen and shows high antioxidant ability. Also, the spiropyridoindolepyrrolidine exhibited antimicrobial ability, and the method that is used for this purpose is the disk diffusion method, and two kinds of bacteria, Gram-positive and Gram-negative, were employed for this analysis. Also, to better understand the reaction mechanism density, functional theory-based quantum chemical methods have been applied. For the generation of spiropyridoindolepyrrolidine, the used process has many properties such as reactions with short time, product with good yields, and simple extraction of catalyst from the mixture of reaction.

## 1 Introduction

**SCHEME 1:**
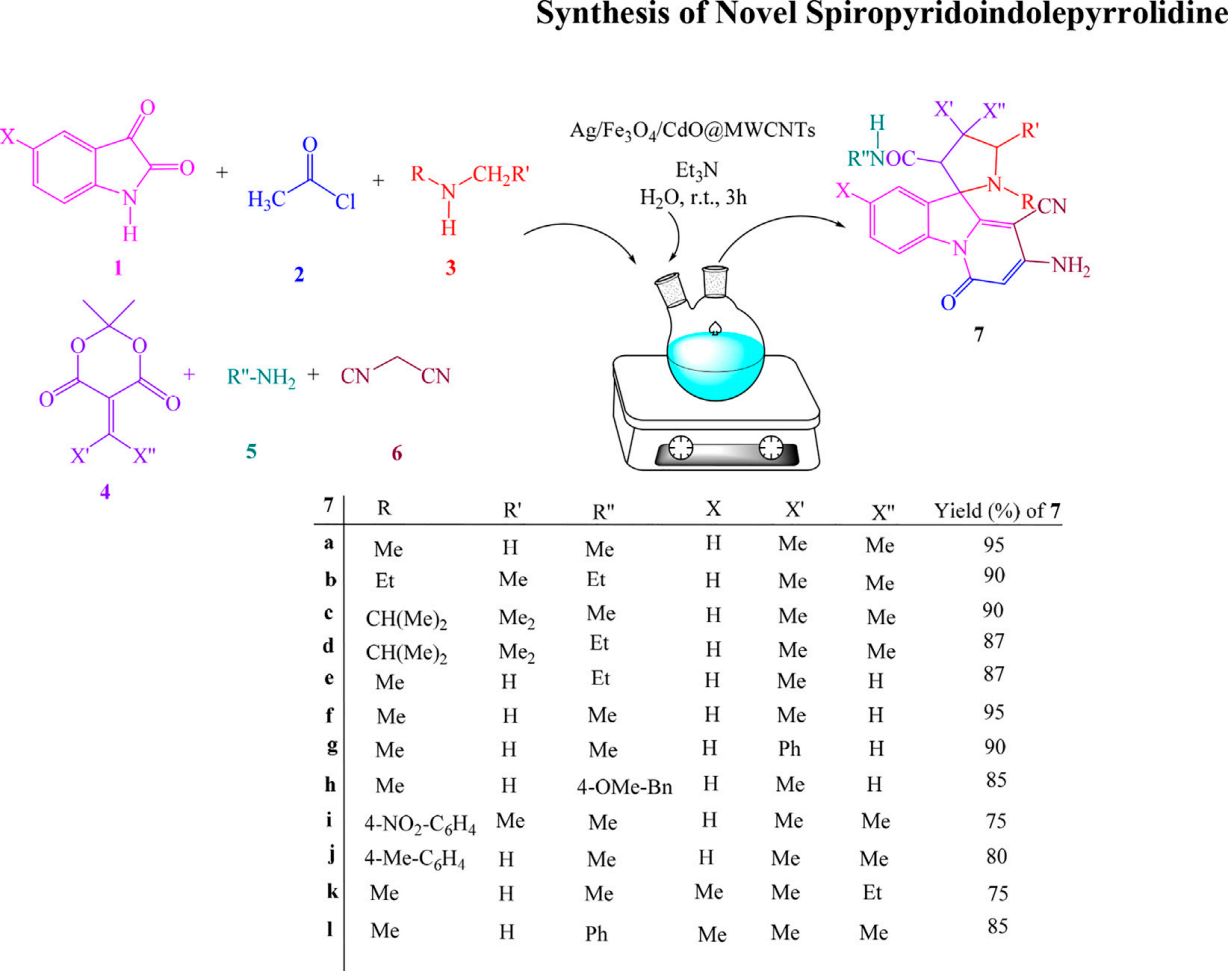
Synthesis of spiropyridoindolepyrrolidines **7.**

Among organic compounds, heterocyclic organic compounds are important because of their application in medicinal chemistry and show many biological activities (Goel et al., 2004; Amir et al., 2007; Abdolmohammadi, et al., 2010; Siddiqui, et al., 2011; Lamberth and Dinges, 2012; Abdolmohammadi, 2013; Abdolmohammadi, et al., 2013; Abdolmohammadi, 2014a; Lashkari, et al., 2015; Martins, et al., 2015; Desai et al., 2016; Zhao et al., 2017; Fouad et al., 2018; Kalaria et al., 2018; Khattab and Rehan 2018; Li et al., 2018; Sokolova et al., 2018; Zhao et al., 2019; Aboonajmi, et al., 2021). Thus, due to the importance of these compounds, many procedures were reported for the synthesis of heterocyclic compounds. One of the most important procedures for the synthesis of these compounds with biological activity is multicomponent reactions (MCRs) (Ibarra, et al., 2018; Zhi et al., 2019). MCRs are significant because they have benefits such as atom economic and synthesis of high-yield heterocyclic compounds in comparison to other procedures (Tietze et al., 2006; Weber, et al., 1999; Herrera and Marqués-López, 2015). Multicomponent reactions (MCRs) are considered to be an important methodological arsenal in synthetic and medicinal chemistry. These reactions have been strategically employed in various synthetic transformations where classical methods usually involve many steps with tedious procedures. The MCR approach provides high yields, atom-/step economy, reduced reaction time, is ecofriendly, and acts as an amenable tool for the generation of a library of new chemical entities (NCEs), especially in the drug discovery process. Extensive research has led to copious developments in the field of MCRs. The developments have emerged with different synthetic approaches, including C–H activation, coupling, and cycloaddition, and eventually, such an amalgamation has enabled access to a broad spectrum of organic frameworks. Some of the procedures for the synthesis of heterocyclic compounds employed the catalyst for performing the reactions. The transition metal oxide nanostructures with high active surface area could be used as catalysts in these reactions. Also, these catalysts are employed in technology and applied science (Sahay et al., 2012; Yang et al., 2019; Zhao et al., 2019; Chen et al., 2020; Yang et al., 2020). MWCNTs, owing to large surface area and high adsorption capability, have been extensively investigated (Zhang, et al., 2013; Khalilian et al., 2017; Samani et al., 2018; Abdolmohammadi, 2018; Abdolmohammadi et al., 2019; Abdolmohammadi et al., 2020b). In the recent past, the supported catalyst and bimetallic oxide or trimetallic oxide catalysts were significant due to their high potentials in carrying out highly selective and efficient organic reactions (Guo et al., 2004; Wachs, 2005; Dastan et al., 2012; Rabiei, et al., 2017; Fakheri-Vayeghan, et al., 2018; Janitabar-Darzi and Abdolmohammadi, 2019; Chaghari-Farahani, et al., 2020; Abdolmohammadi, et al., 2022; Zare Davijani et al., 2022a). Metal oxides have high-crystalline structure and catalytic efficiency (Shi, 2013; Mousavi, et al., 2014; Jabłonska and Palkovits, 2016; Sharghi, et al., 2018). For this reason, the mixture of two or more metals and their curing procedures permit the change of the properties of materials’ surface and optimization of the properties for a particular goal (Zhang et al., 2012; Lin-Bing et al., 2015). Therefore, the mixture of metal oxide catalysts and nanocomposite structures of them exhibited production of heterocyclic compounds according to green rules with high efficiency (Abdolmohammadi, et al., 2019; Abdolmohammadi et al., 2020a; Kalantari et al., 2020; Abdolmohammadi, et al., 2021; Khalilzadeh et al., 2021). Among the metal oxide nanoparticles, Fe_3_O_4_ magnetic nanoparticles (MNPs) are important because of high surface area, simple removing from reaction, and employment of it in MCRs several times. Another subject that is investigated in this research is the study of biological abilities such as the antioxidant and antimicrobial activity of synthesized spiropyridoindolepyrrolidines. Compounds with antioxidant activity could eliminate the negative effect of free radicals due to having reduction chemical structure. Also, these compounds could be employed as transitional metals chelators, and many sicknesses could be decreased or treated by these compounds (Halliwell, 1999; Liu and Meydani, 2002; Babizhayev et al., 2004; Djurišić et al., 2012). Another investigation of biological activity is the antimicrobial ability of synthesized compounds. Some of the bacteria do not get killed by utilizing drugs, and these bacteria cause many diseases in humans and animals. For this reason, finding out good yielding procedures for decreasing this problem and investigation of the antimicrobial properties of synthesized compounds are important. Dyes and pigments are two important subjects that are used in the generation processes of food, drug, textile, and printing. The production of dyes and pigments is about ∼7 × 105 tons in one year, and these are very unsafe and dangerous for aquatic system organisms (Kassaee, et al., 2004; Yavari, et al., 2007; Yavari et al., 2010; Ezzatzadeh and Hossaini 2019). For this reason, discovering green and eco-friendly procedures for removing dye and pigment pollutants from the environment is very important. Many of these procedures that are investigated in the literature produced a byproduct, and for this reason, they are not suitable for usage. Also, recently, the application of nanotechnology in water treatment has gained wide attention and is being actively investigated due to its remarkable properties (Kassaee et al., 2008; Sabbaghan et al., 2010; Rustaiyan and Ezzatzadeh, 2011; Ezzatzadeh et al., 2012; Shahvelayati and Esmaeeli, 2012; Zhang et al., 2013; Salehi Borban et al., 2017; Shahvelayati et al., 2017; Seifi Mansour et al., 2019; Ghambarian et al., 2020; Ghashghaee and Ghambarian, 2020; Shafaei et al., 2020). In the field of water treatment, nanotechnology can be classified into three main applications: 1) to restore (remediate) and purify contaminated water, 2) to detect pollution, and 3) to prevent pollution. This has led to the demand for nanosensors with high sensitivity for the detection of micro-, nano-, and molecular pollutants. Therefore, high-efficiency methods or actively synthesized compounds were needed for eliminating or decreasing these problems. In recent years, we studied about enhancing new and easy processes for the generation of important heterocyclic compounds (Babizhayev et al., 2004; Ebrahimi, et al., 2008; Kassaee et al., 2010; Yavari et al., 2010; Khandan-Barani et al., 2011; Rustaiyan, et al., 2011; Rustaiyan, et al., 2011; Shahvelayati et al., 2012; Ezzatzadeh et al., 2012; Hassanabadi and Khandan-Barani, 2013; Masoodi, et al., 2013; Abdolmohammadi, 2014b; Maghsoodlou, et al., 2014; Azizi et al., 2015; Hajinasiri et al., 2015; Khandan-Barani et al., 2015; Balar et al., 2016; Balar et al., 2016; Ghambarian et al., 2016; Soleimani Amiri, et al., 2016; Ghambarian et al., 2017; Koohi, et al., 2017; Salehi Borban et al., 2017; Shahvelayati et al., 2017; Soleimani-Amiri et al., 2018; Abdolmohammadi and Hossaini, 2019; Ghashghaee et al., 2019; Hekmatara et al., 2019; Mohammadi, et al., 2019; Seifi Mansour et al., 2019; Abdolmohammadi et al., 2020a; Ghashghaee et al., 2020a; Ghashghaee et al., 2020b; Shafaei et al., 2020; Ghavidel, et al., 2021; Zare Davijani et al., 2022b). In this research, initially, a green procedure was employed for the generation of new spiropyridoindolepyrrolidines **7** *via* MCRs of isatins **1**, acetyl chloride **2**, secondary amines **3**, vinilidene Meldrum’s acid **4**, primary amines **5,** and malononitrile **6** in an aqueous medium at room temperature in the vicinity of Ag/Fe_3_O_4_/CdO@MWCNT MNCs as an organometallic catalyst in aqueous media at room temperature (Scheme 1).

## 2 Results and discussion

**FIGURE 1:**
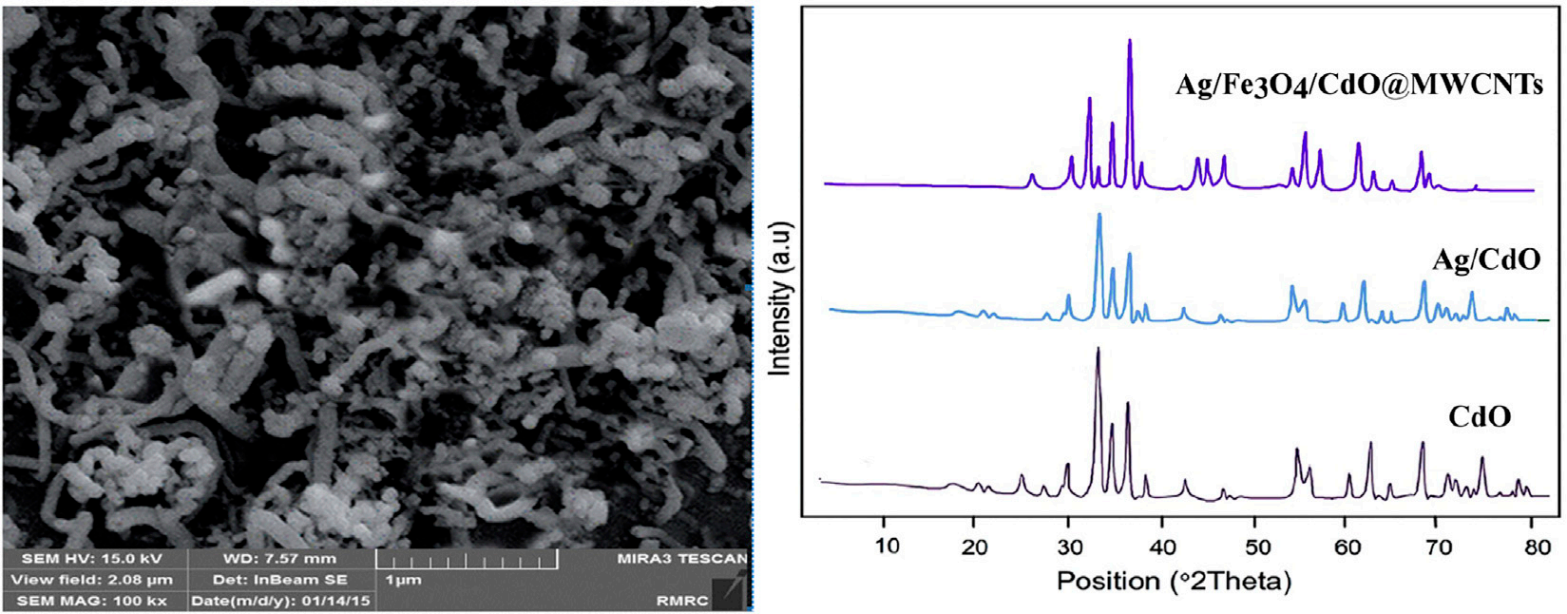
Left: SEM image of Ag/Fe_3_O_4_/CdO@MWCNT MNCs. Right: the XRD analysis of Ag/Fe_3_O_4_/CdO@MWCNT MNCs.

In this study, the new spiropyridoindolepyrrolidines **7** have been produced with excellent efficiency employing six component reactions of isatins **1**, acetyl chloride **2**, secondary amines **3**, vinilidene Meldrum’s acid **4**, primary amines **5,** and malononitrile **6** in aqueous media at room temperature in the vicinity of Ag/Fe_3_O_4_/CdO@MWCNT MNCs as a new reusable organometallic nanocatalyst. The water extract of *Petasites hybridus* rhizome was used for the preparation of Ag/Fe_3_O_4_/CdO@MWCNT MNCs, and for the confirmation of the prepared catalyst structure, scanning electron microscopy (SEM) (Figure 1 (left)) and X-ray diffraction (XRD) (Figure 1 (right)) were employed. The SEM image of Ag/Fe_3_O_4_/CdO@MWCNT MNCs was used for the investigation and confirmation of the skeleton of the organometallic nanocomposite. The morphology, surface uniformity, and size of particles can be investigated by SEM analysis. The SEM images of Ag/Fe_3_O_4_/CdO and Ag/Fe_3_O_4_/CdO@MWCNT magnetic nanocomposites are shown in Figure 1 (left). Based on the FE-SEM images, Ag/Fe_3_O_4_/CdO MNCs have a spherical morphology, which is confirmed by the good dispersion of nanoparticles in the structure.

Another analysis for the confirmation of the structure of the nanocatalyst is X-ray diffraction (XRD) which was utilized for measuring the size of synthesized Ag/Fe_3_O_4_/CdO@MWCNT MNCs (Figure 1 (right)). All of the observed peaks at 2θ = 35.0, 44.0, 54.2, 57.2, and 63.0° can be contributed to Fe_3_O_4_ (JCPDS No. 19-629) that have the face-centered cubic phase. All peaks are for the pure Fe_3_O_4_, and the structure of Fe_3_O_4_ did not display any impurity. In the XRD analysis of synthesized Ag/Fe_3_O_4_/CdO@MWCNT, MNCs showed peaks at 2θ = 33.9, 54.8, and 68.2^o^ that could be contributed to (111), (311), and (222) planes and show the cubic construction for CdO moiety (JCPDS No: 73-2,245). The peaks in the XRD analysis of the synthesized catalyst that are attributed to Ag NPs are at 37.9, 44.6, 64.6, and 77.7^o^ and (111), (200), (220), and (311) planes of Ag (JCPDS, No.04-0783), respectively, and the Ag_2_O peak was not seen in XRD analysis. The typical peaks of MWNTs moiety (JCPDS No. 41-1,487) were seen as broad peaks around 26.2 and 43.6^o^.

**FIGURE 2:**
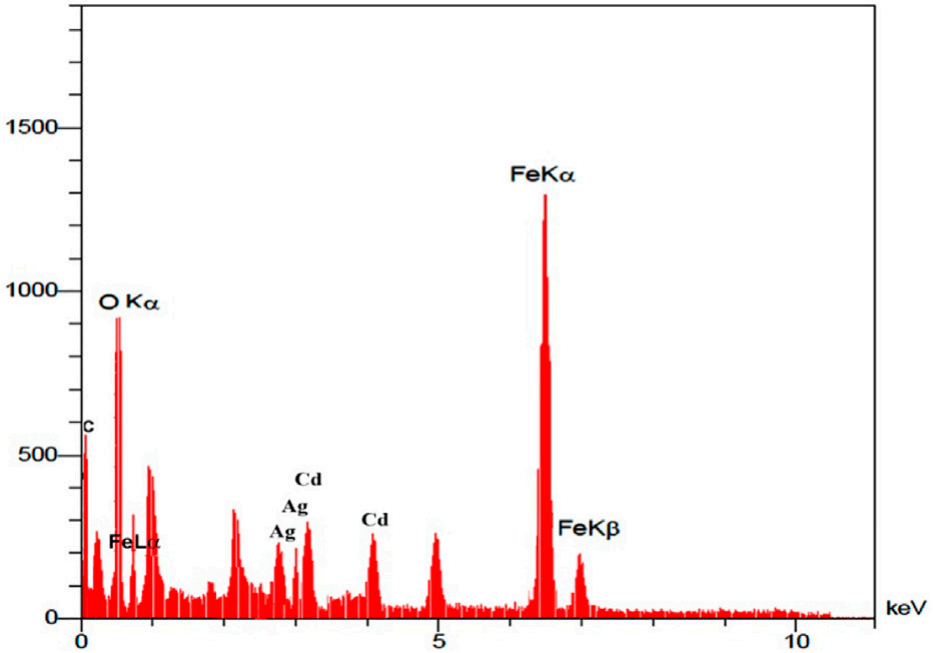
EDX image of Ag/Fe_3_O_4_/CdO@MWCNT MNCs.

The elemental analysis of the synthesized nanocatalyst was specified by the Energy Dispersive X-Ray Analysis (EDX) procedure (Figure 2). The peaks that are seen in the EDX analysis of Ag/Fe_3_O_4_/CdO@MWCNT are Ag, Cd, Fe, O, and C peaks which confirmed the production of nanocatalysts. Additionally, the existence of peak of carbon confirmed that the organic compounds and multi-walled carbon nanotubes (MWCNTs) exist in the synthesized nanocatalyst.

**FIGURE 3:**
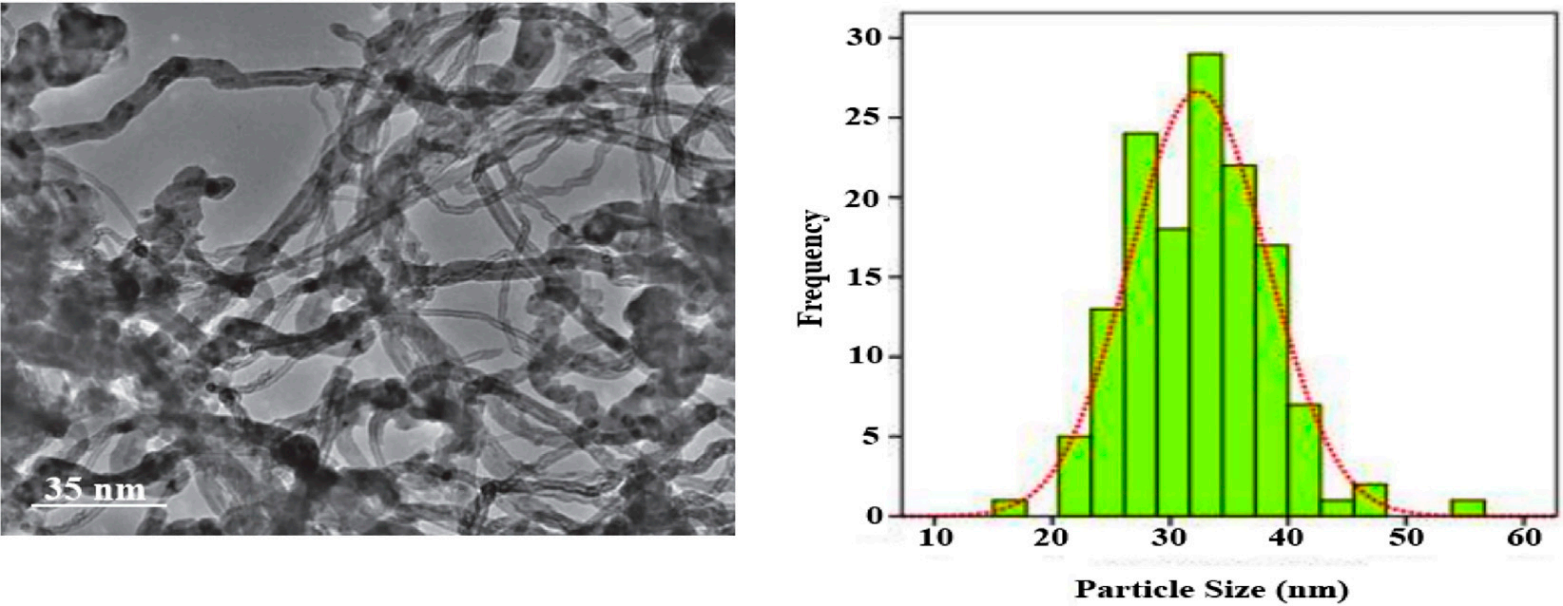
TEM image and histogram curve of Ag/Fe_3_O_4_/CdO@MWCNT MNCs.

In another technique for confirming the morphology of the Ag/Fe_3_O_4_/CdO@MWCNT, MNCs were considered by transmission electron microscopy (TEM) (Figure 3). Figure 3 (left) gives the TEM micrographs of clean Ag/Fe_3_O_4_/CdO@MWCNTs, and Figure 3 (right) gives the histogram curve of Ag/Fe_3_O_4_/CdO@MWCNT MNCs.

**FIGURE 4:**
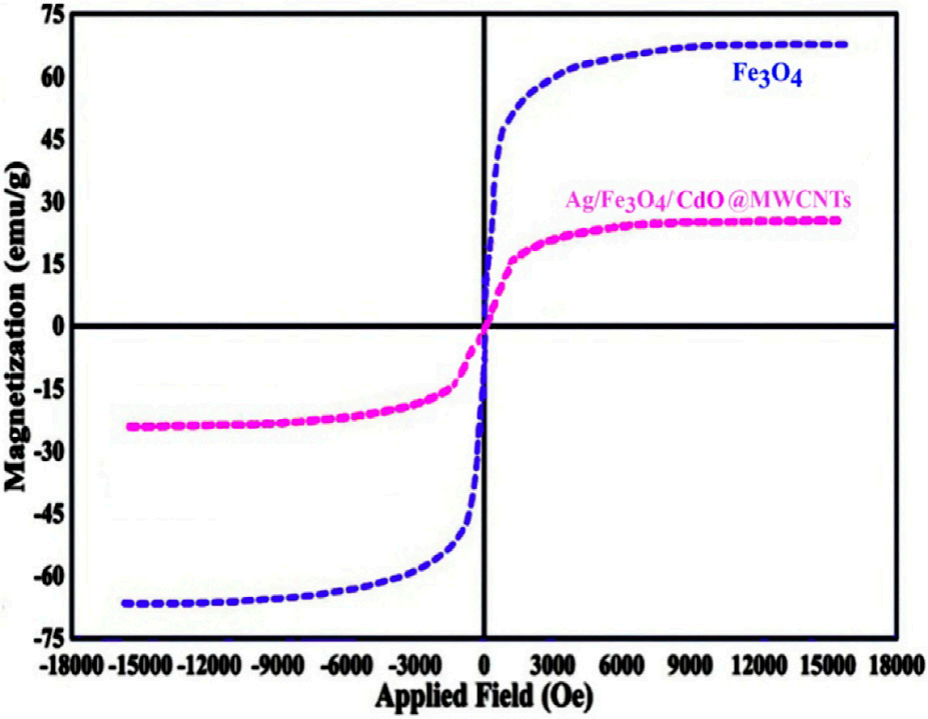
VSM analysis of the green Ag/Fe_3_O_4_/CdO@MWCNT MNCs.

To determine the mean size of the Ag/Fe_3_O_4_/CdO@MWCNTs nanoparticles, we obtained the histogram (by measuring about 100 randomly chosen particles in the magnified TEM image) of the particle size distribution of Ag/Fe_3_O_4_/CdO@MWCNTs (Figure 3 (right)). The graphical results in this figure show that the size of the Ag/Fe_3_O_4_/CdO@MWCNT nanoparticles is in the range of 10–60 nm with the average particle size of 33 nm. The magnetic property of the synthesized nanocatalyst and pure Fe_3_O_4_ NPs was confirmed by measuring the value of saturation magnetization (VSM) Figure 4. According to the VSM analysis of nanocatalyst, the Ag/Fe_3_O_4_/CdO@MWCNT MNCs’ and pure Fe_3_O_4_ NPs’ saturation magnetization is 28.3 emu/g and 67.5 emu/g, respectively (Figure 4). Because of the catalyst structure and coating effect of the synthesized catalyst, the magnetic response was decreased. The magnetic property of the synthesized catalyst caused easy separation of the catalyst from the aqueous solution.

We also evaluated the catalytic activities of the Ag/Fe_3_O_4_/CdO@MWCNT MNCs through the synthesis of spiropyridoindolepyrrolidine derivatives in the presence of Ag/Fe_3_O_4_/CdO@MWCNT MNCs. The imperative subject in all organic reactions is obtaining the most excellent state for carrying out the reactions. In the beginning, for obtaining this goal, the multicomponent reaction of isatins **1a**, acetyl chloride **2**, dimethyl amine **3a**, isopropylene Meldrum’s acid **4a**, methyl amine **5a,** and malononitrile **6** was chosen as a model reaction (Table 1). Without a catalyst, even after 10 h, the production of compound **7a** was not carried out (entry 1, Table 1). For optimizing the temperature of sample reaction and achieving the best temperature, the reaction temperature was enhanced to 100^o^C but did not exhibit significant variation in the efficiency of spiropyridoindolepyrrolidine **7a** (entry 2, Table 1). Also, these reactions were not carried out without a catalyst. For confirmation of this point, to the reaction mixture, CdO-NPs (0.01 g) were added as catalysts. After 4 h, spiropyridoindolepyrrolidine **7a** was generated in good efficiency (entry 4, Table 1). As a result, these reactions needed a catalyst for performing. For finding out the best catalyst for the model reaction, we considered a number of nanocatalysts such as Ag NPs, Fe_3_O_4_ MNPs, CdO NPs, Fe_3_O_4_/CdO NPs, Fe_3_O_4_/CdO/MWCNTs, Ag@MWCNTs, MWCNTs, Ag NPs, and Ag/Fe_3_O_4_/CdO@MWCNTs. Among these catalysts, Ag/Fe_3_O_4_/CdO@MWCNTs are selected as nanocatalysts for the synthesis of spiropyridoindolepyrrolidine **7a,** and the product efficiency was increased by this catalyst. The Ag/Fe_3_O_4_/CdO@MWCNTs as catalysts have two sites in their structure. The three sites in the synthesized catalyst (Ag, Fe, and Cd) are Lewis acids and caused the activation of carbonyl groups. According to the results in Table 1, the Ag NPs are more useful than Fe_3_O_4_, CdO, Fe_3_O_4_/CdO, and Fe_3_O_4_/CdO/MWCNT. Ag is a stronger Lewis acid than Fe_3_O_4_ and TiO_2_ and is very significant when these reactions were performed with catalytic amounts of Ag/Fe_3_O_4_/CdO/MWCNTs-MNCs. Therefore, as a result, increasing the Ag/Fe_3_O_4_/CdO/MWCNTs-MNCs’ amount from 0.02 to 0.03 g did not illustrate any remarkable variation in the efficiency of the reaction. So, 0.02 g of Ag/Fe_3_O_4_/CdO/MWCNTs-MNCs was needed for the preparation of spiropyridoindolepyrrolidine with high efficiency (entry 11, Table 1), and the yield of compound **7a** is 95% after 3 h (entry 11, Table 1). The roles of nanocatalysts in the preparation of spiropyridoindolepyrrolidine derivatives are Lewis acid and Lewis base. Ag, Cd, and Fe as Lewis acid activate the carbonyl group for nucleophilic attack. As displayed in Table 1, among Lewis acids, Ag is more effective than Cd and Fe.

**TABLE 1:** Determination of the best conditions such as the catalyst, amount of catalyst, and temperature for the synthesis of **7a**.

Entry	Catalyst	Temp. (°C)	Catalyst (g)	Time (h)	Yield %^a^
1	None	r.t	-	10	-
2	None	100	-	8	-
3	CdO-NPs	r.t	0.01	4	45
4	CdO-NPs	r.t	0.015	3	58
5	CdO-NPs	r.t	0.02	3	65
6	Fe_3_O_4_-MNPs	r.t	0.015	3	35
7	Ag NPs	r.t	0.015	3	78
8	MWCNT	r.t	0.015	3	27
9	CuO-NPs	r.t	0.015	3	38
10	Ag/Fe_3_O_4_/CdO	r.t	0.015	3	80
11	Fe_3_O_4_/CdO	r.t	0.015	3	70
12	Ag/CdO@ MWCNT	r.t	0.015	3	78
13	Fe_3_O_4_/CdO/MWCNT	r.t	0.015	3	70
14	CdO@ MWCNT	r.t	0.015	3	68
15	Ag/Fe_3_O_4_/MWCNT	r.t	0.015	3	75
16	Ag/Fe_3_O_4_/CdO@MWCNT	r.t	0.015	3	87
**17**	**Ag/Fe**_**3**_ **O**_**4**_ **/CdO@MWCNT**	**r.t**	**0.02**	**3**	**95**
18	Ag/Fe_3_O_4_/CdO@MWCNT	reflux	0.02	3	95
18	Ag/Fe_3_O_4_/CdO@MWCNT	r.t	0.015	3	87
19	Ag/Fe_3_O_4_/CdO@MWCNT	r.t	0.025	3	95

a Isolated yields. Bold indicates best condition for performing reactions.

Because of the easy and simple extraction of Fe_3_O_4_ magnetic nanoparticles (MNPs) from the reaction mixture, employing it in several reactions is very significant. Additionally, another study in this research work is the investigation of solvents’ effects that were investigated on the synthesis of compound **7a** in the presence of Ag/Fe_3_O_4_/CdO@MWCNT (0.02 g). The results in Table 2 display that for carrying out the reaction, water is the best solvent.

**TABLE 2:** Determination of the best solvent for the generation of **7a**.

Entry	Solvent	Time (h)	Yield %^a^
1	EtOH	15	None
2	CH_2_Cl_2_	8	60
3	CHCl_3_	5	68
**4**	**H**_**2**_ **O**	**3**	**95**
5	Solvent free	8	58
6	DMF	12	30
7	Toluene	12	68
8	CH_3_CN	5	90

a Isolated yields.

Bold indicates best condition for performing reactions.

As illustrated in Table 1 and Table 2, Ag/Fe_3_O_4_/CdO@MWCNT-MNC (0.02 g) as an organometallic catalyst, room temperature, and aqueous media are the suitable conditions for the generation of spiropyridoindolpyrrolidine **7**. The reuse of the synthesized catalyst is an important factor in the synthesis of organic compounds. In this research, the synthesized nanocatalyst was reused five times for the synthesis of spiropyridoindolepyrrolidines **7a** (Table 3). The obtained results proved that the catalyst can be reused five times without any considerable change in its power (Table 3). For reusing magnetic nanocatalysts, the external magnet was needed for the separation of the catalyst from the reaction mixture. After separation, the catalyst was washed with water and dried at room temperature for 24 h and used again.

**TABLE 3:** Number of reusability of the catalyst for the synthesis of compound **7a**.

Run	% yield^a^
1st	95
2nd	95
3rd	92
4th	90
5th	87

a Isolated yields.

**SCHEME 2:**
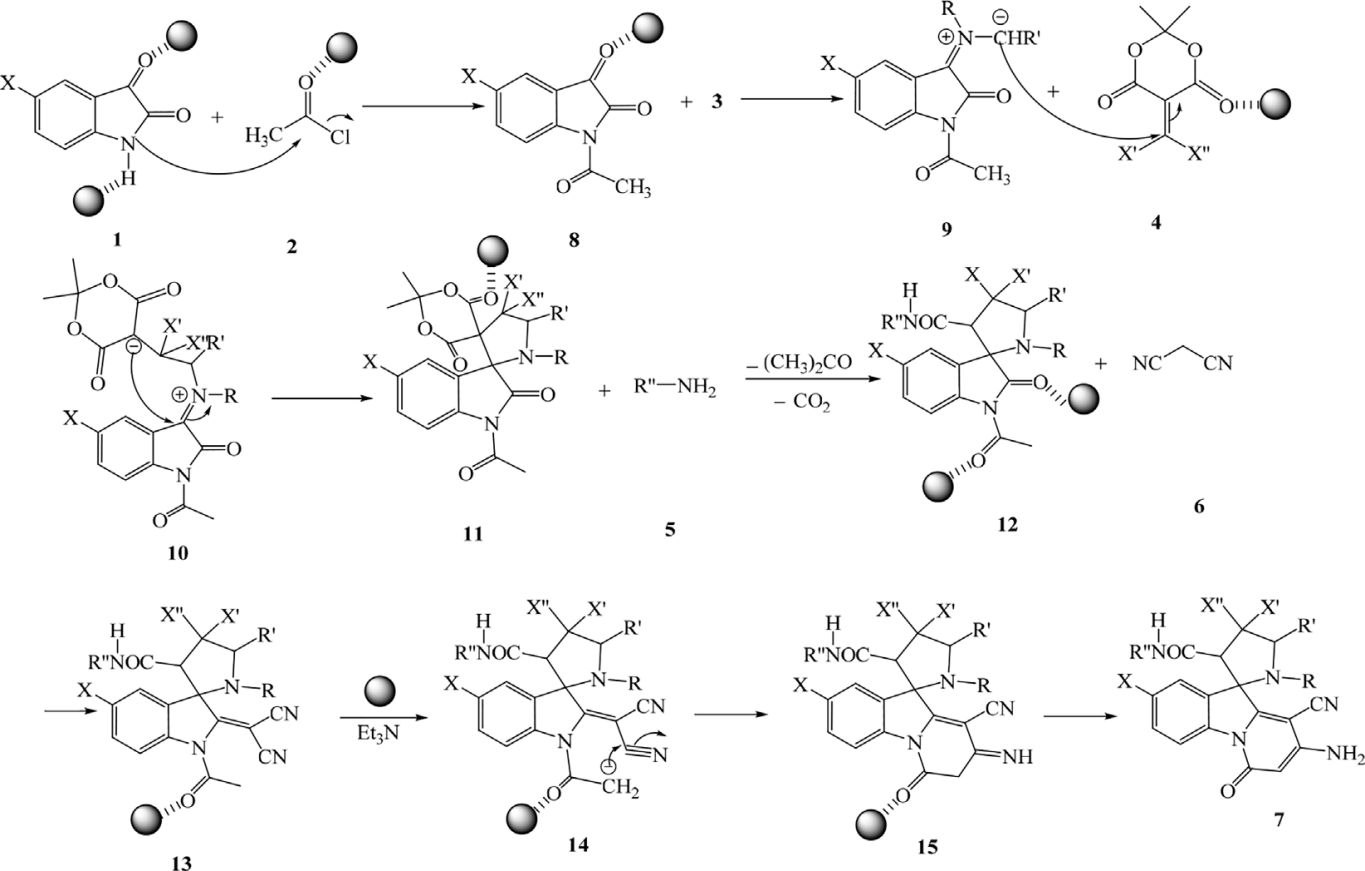
Proposed mechanism for the preparation of **7.**

After each run for the synthesis of compound **7a**, the catalyst was removed from the reaction mixture, washed, and used again. For this reason, compound **7a** yield was reduced after five times due to the decrease in the amount of catalyst and separation of it after each run. It should be mentioned that after separation of the catalyst, the amount of catalyst maybe changed but the form and size of it were not altered after separation. The decrease in the amount of catalyst has the highest effect on the efficiency of compound **7a**. For confirming the structure of synthesized spiropyridoindolepyrrolidines **7**, ^1^H NMR, ^13^C NMR, IR, elemental analysis, and mass spectrum were employed. At 1.05 and 1.12 ppm in the ^1^H NMR spectra of spiropyridoindolpyrrolidine **7a** were displayed two singlets for methyl protons. There were two singlets at 2.25 and 2.76 ppm for NMe protons, one singlet at 3.64 for CH proton, one singlet at 9.87 for NH and at 10.23 for NH_2_ protons, and several signals for aromatic protons. The carbonyl moiety displays two resonances in the ^13^C NMR spectra of **7a** at 164.8 and 173.5 ppm. Also, another route for confirming the existence of carbonyl groups in the construction of synthesized compounds is the IR spectrum. The preparation mechanism for the synthesized compounds **7** is shown in Scheme 2 (Sabbaghan, et al., 2010).

First, isatins **1** and acetyl chloride **2** react at room temperature in the presence of Ag/Fe_3_O_4_/CdO@MWCNT MNCs and intermediate **8** was generated. The secondary amines **3** react with the carbonyl group of intermediate **8** and produced the iminium ion **9** that with react with vinilidene Meldrum’s acid **4** and produced intermediate **10**. Intermolecular cyclization of intermediate **10** produced intermediate **11** that was reacted with amines **5** and generated compounds **12** by the elimination of acetone and carbon dioxide. Compounds **12** react with malononitrile in the presence of catalyst and with intermolecular cyclization produced spiropyridoindolepyrrolidines **7**.

**SCHEME 3:**
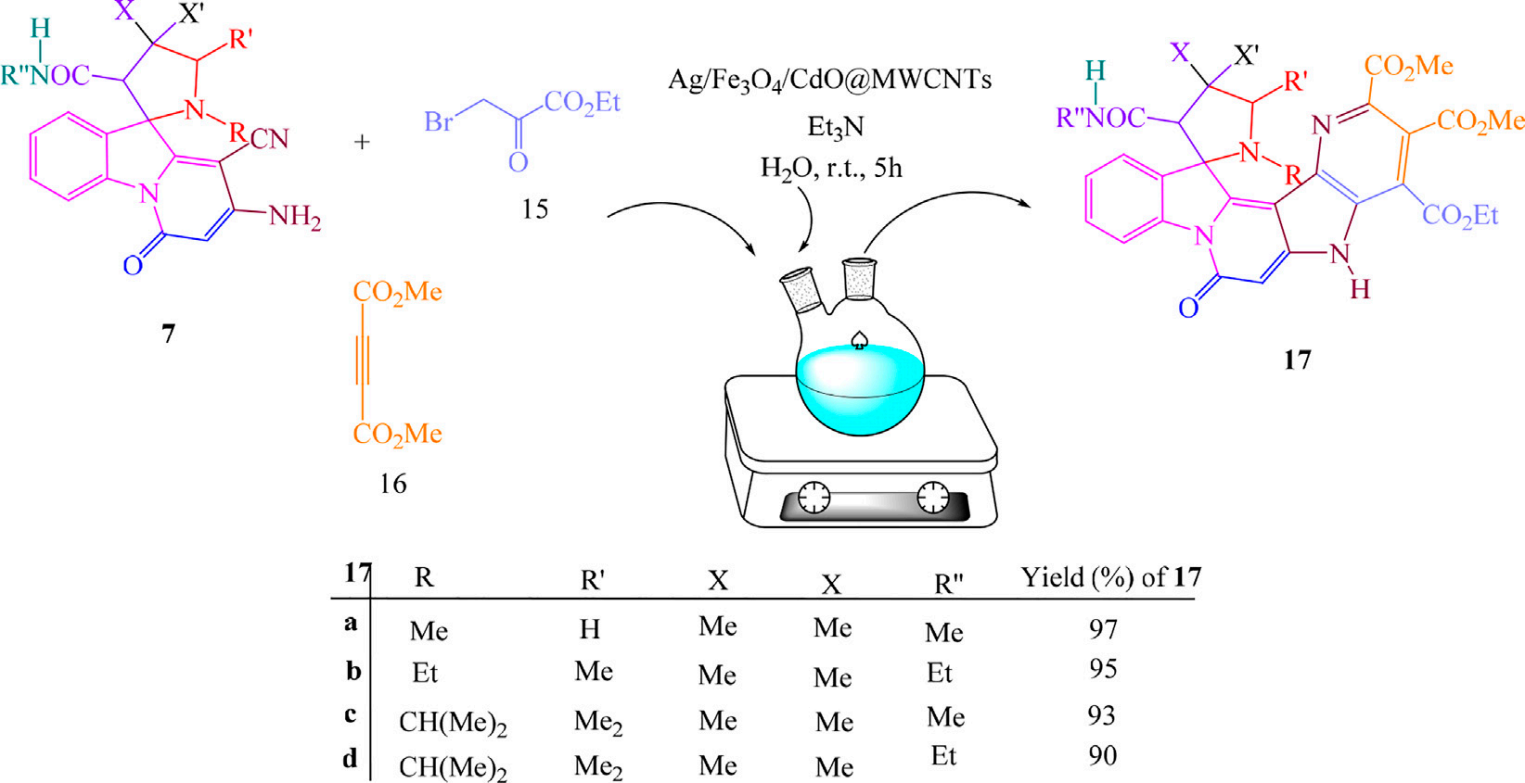
Synthesis of spiropyridines **17.**

Under similar conditions and for confirming the structure of compounds **7**, the multicomponent reactions of spiropyridoindolepyrrolidines **7**, ethyl bromopyruvate **15,** and dimethyl acetylenedicarboxylate **16** in the vicinity of Ag/Fe_3_O_4_/CdO@MWCNT MNCs as an organometallic catalyst were performed in aqueous media at room temperature and produced spiropyridines 17 (Scheme 3).

**SCHEME 4:**
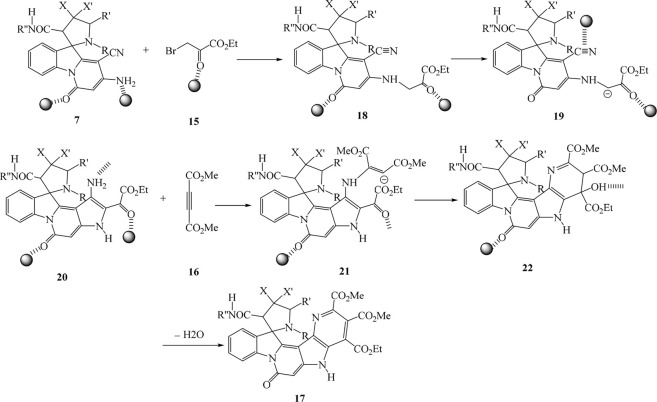
Proposed mechanism for the preparation of 1**7.**

For confirming the structure of compounds IR, ^1^H NMR, and ^13^C NMR, mass spectra were employed. At 1.05 and 1.10 ppm in the ^1^H NMR of spiropyrrolopyridine **17a,** two singlets were displayed for methyl protons. The singlet at 2.76 related to NMe protons, the singlet at 3.07 attributed to CH_2_ protons, and also, the singlets in the 3.61, 3.84, and 3.91 attributed to CH and two methoxy protons. One singlet at 5.15 ppm is for CH protone, and two singlets at 7.52 and 11.34 are for NH protones with aromatic protons resonances. The resonances at δ 165.2, 166.6, 168.8, and 172.1 ppm were contributed to four carbonyl groups of 1**7a**. Also, the carbonyl moiety in the structure of **17a** was confirmed by giving the IR spectrum**.** The proposed mechanism was recommended for the synthesis of compounds **17** as in Scheme 4.

### Newly synthesized organometallic catalyst promoted the reduction of the organic pollutant

**FIGURE 5:**
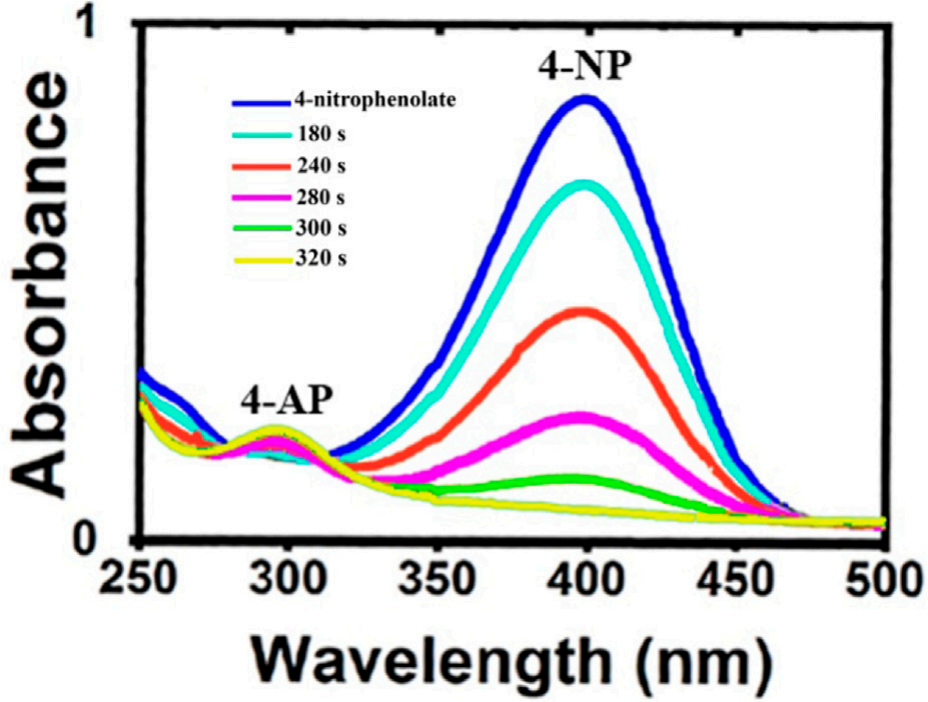
Reduction of the 4-NP to 4-AP by UV–Vis analysis.

Another subject in this research is the investigation of the organometallic nanocatalyst effect on the elimination of the organic pollutant such as 4-nitrophenol. The effect of prepared Ag/Fe_3_O_4_/CdO@MWCNT MNCs on the reduction of 4-NP as a high-performance organometallic catalyst was studied in water at room temperature. The dye reduction processes were confirmed by giving the UV–Vis spectrum from the reaction mixture at ambient temperature. The reduction of 4-NP was not performed in the absence of the catalyst and NaBH_4_ and did not display significant consideration. Therefore, Ag/Fe_3_O_4_/CdO@MWCNT MNCs were needed for the reduction of 4-NP to 4-aminophenol. The variation of reaction conditions monitored by UV–Vis analysis is exhibited in Figure 5. In the UV–Vis spectrum, the peak at 317 nm was contributed to the absorption of 4-nitrophenol in water, but when 4-nitrophenol converted to 4-nitrophenolate ion, the peak at 317 nm shifted to 400 nm as a red shift. After adding the newly prepared aqueous solution of NaBH_4_, the color changed from yellow to colorless. If the solution was not added to Ag/Fe_3_O_4_/CdO@MWCNTs, the peak that was seen at 400 nm was not altered even after 15 h, which confirmed that reduction of 4-NP was not carried out in the absence of catalyst Ag/Fe_3_O_4_/CdO@MWCNTs with NaBH_4_ and vice versa. When Ag/Fe_3_O_4_/CdO@MWCNTs were in 4-nitrophenol solution without NaBH_4_, any considerable change in the color was not seen. For the elimination of the organic pollutant, the prepared organometallic nanocatalyst and NaBH_4_ were needed, and in the presence of the catalyst and reducing agent, the peak of absorbance at 400 nm quickly within 5 min reduced to about zero. A new peak was generated after reducing at 300 nm that attributed to the formation of 4-aminophenol and regularly enhanced to lighten the yellow color of the reaction solution.

Furthermore, for the reusability of the synthesized catalyst, the external magnet was used and separated the magnetic catalyst completely from the reaction mixture without any variation in catalytic activity. The advantages of this method for the reduction of the organic pollutant and employing the organometallic biocatalyst Ag/Fe_3_O_4_/CdO@MWCNTs are the effectiveness, ecological type, and inexpensive process for the removal and photo decreasing of 4-NP.

### Evaluation of the antioxidant property of prepared spiropyridoindolpyrrolidines by DPPH

**FIGURE 6:**
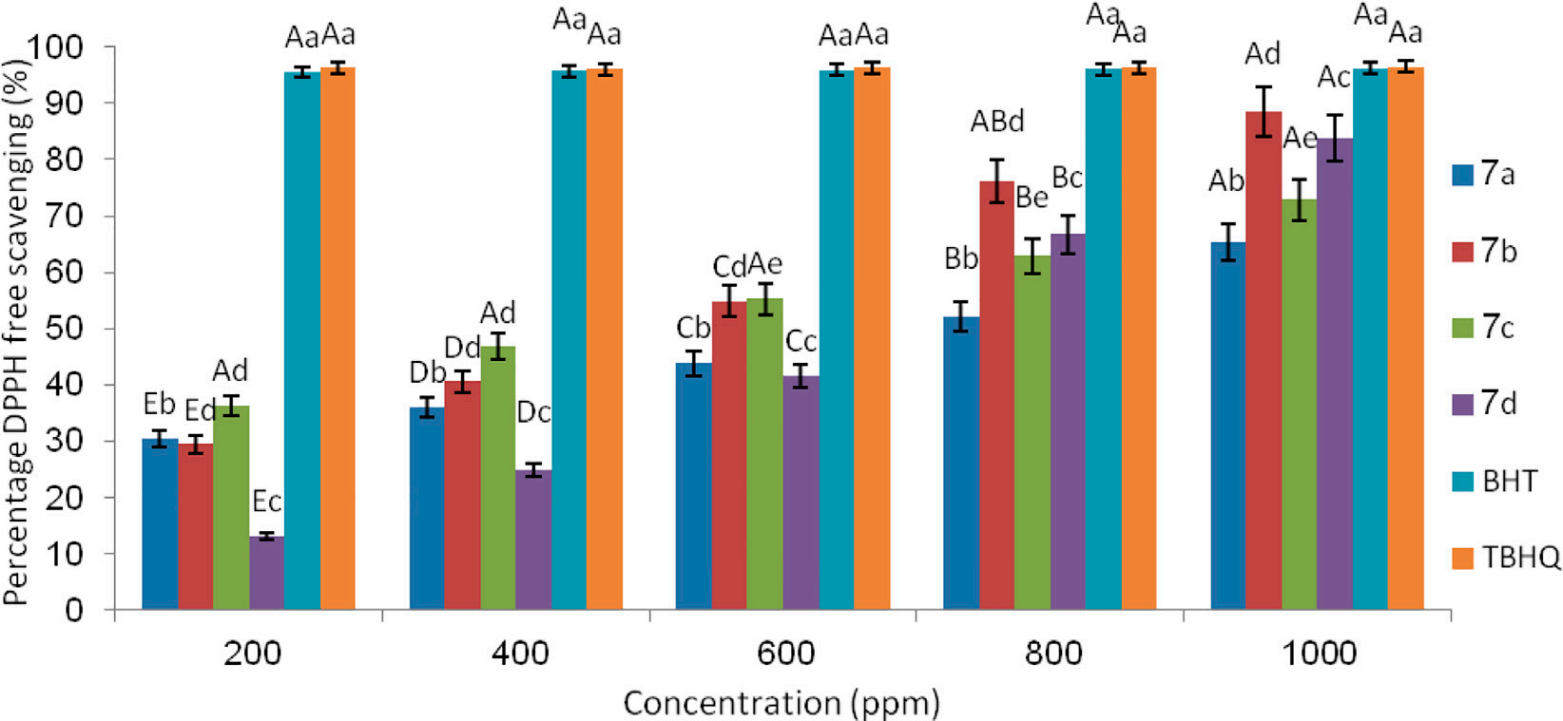
Order of antioxidant activity of **7a-7d** using DPPH.

Another purpose of this research is the study of the antioxidant property of synthesized spiropyridoindolpyrrolidines, and for achieving to this goal, DPPH was used. It should be mentioned that the DPPH radical scavenging test was employed for many purposes such as for studying the antioxidant activity of synthesized organic compounds, foods, and biological structures (Ahmadi et al., 2007; Bidchol et al., 2011; Saundane and Nandibeoor, 2015) *via* taking an electron or hydrogen atom by the free radical of DPPH. The synthesized spiropyridoindolepyrrolidine loses the hydrogen atom or one electron in the presence of the DPPH radical, and this means that these compounds have antioxidant property. The percentage of trapping the DPPH free radical by the synthesized spiropyridoindolpyrrolidines displays the order of antioxidant activity. In this research, we investigated the antioxidant activity of some synthesized compounds such as **7a-7d** and compared to standard synthesized antioxidant BHT and TBHQ, in which the electron or hydrogen absorbance of these compounds by DPPH free radical proved the antioxidant activity of them. When one electron or hydrogen atom is adsorbed by DPPH, the absorbance of it decreases to 517 nm. Overall, the antioxidant ability of spiropyridoindolepyrrolidine derivatives **7a-7d** was achieved as TBHQ ˜ BHT>**7b** > **7d**> **7c** > **7a** (Figure 6).

Figure 8 exhibited that good differences existed between spiropyridoindolepyrrolidines concentration to BHT and TBHQ as the standard antioxidant. Compound **7b** showed good ability relative o BHT and TBHQ among the experimented spiropyridoindolepyrrolidines **7a-7d**,

### Assessment of spiropyridoindolepyrrolidines’ antioxidant activity by Fe^3+^ reduction

**FIGURE 7:**
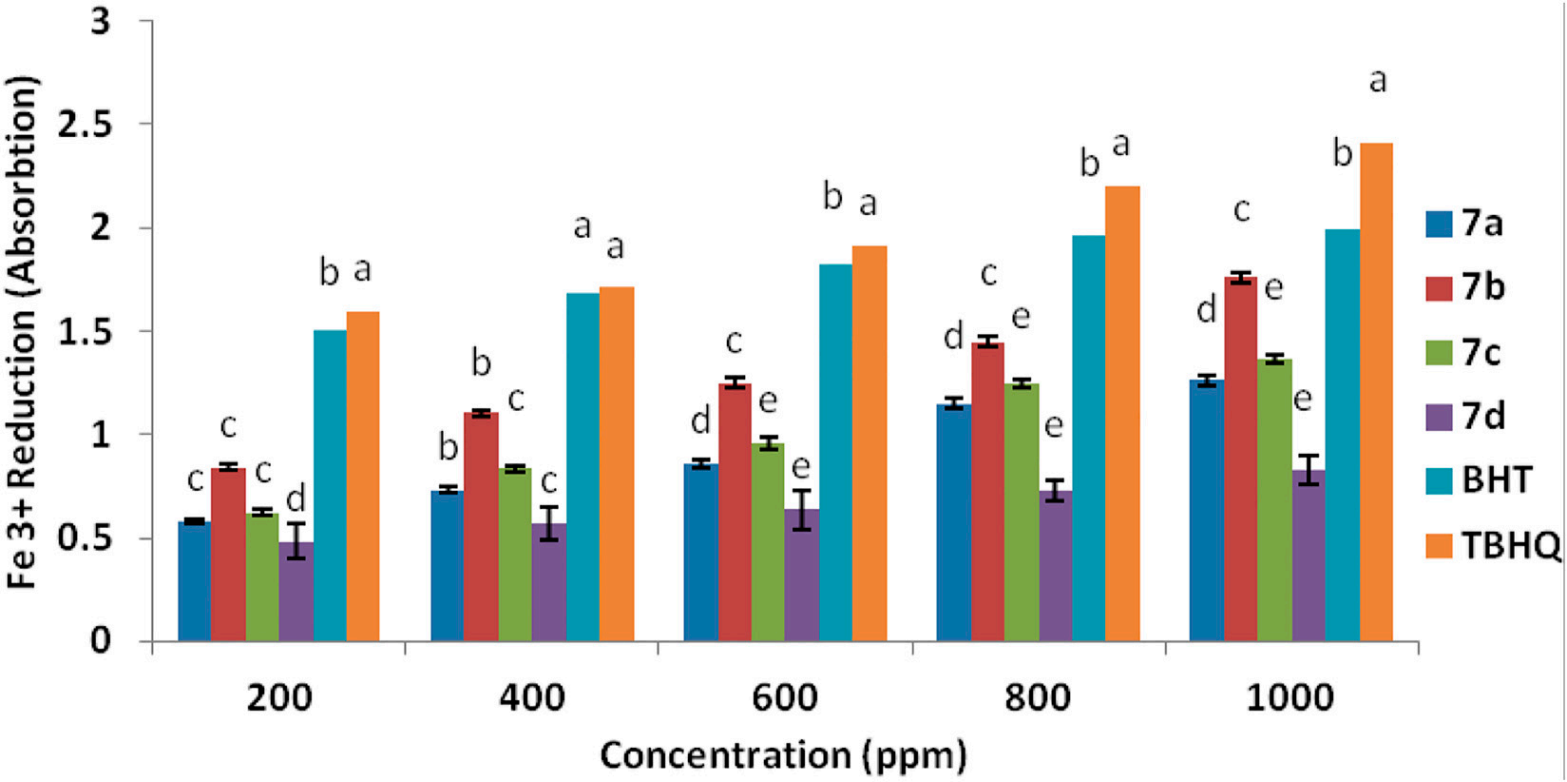
Ferric ions (Fe^3+^) reducing antioxidant power (FRAP) of compounds **7a-7d**.

The antioxidant property of spiropyridoindolepyrrolidines **7a-7d** was tested by another procedure for confirming it. The spiropyridoindolepyrrolidines caused the reduction of ferric ions (Fe^3+^), and the amounts of reduction are measured based on the reduction of Fe^3+/^ferricyanide to the Fe^2+^/ferrous at 700 nm (Yildirim et al., 2001), and spiropyridoindolpyrrolidines **7b** exhibited good effect than BHT and TBHQ. Figure 7 shows the order of the antioxidant activity of spiropyridoindolepyrrolidines **7a-7d** as TBHQ > BHT>**7b** > **7a**>**7c** > **7d**.

### Antibacterial activity evaluation of synthesized spiropyridoindolepyrrolidines

**FIGURE 8:**
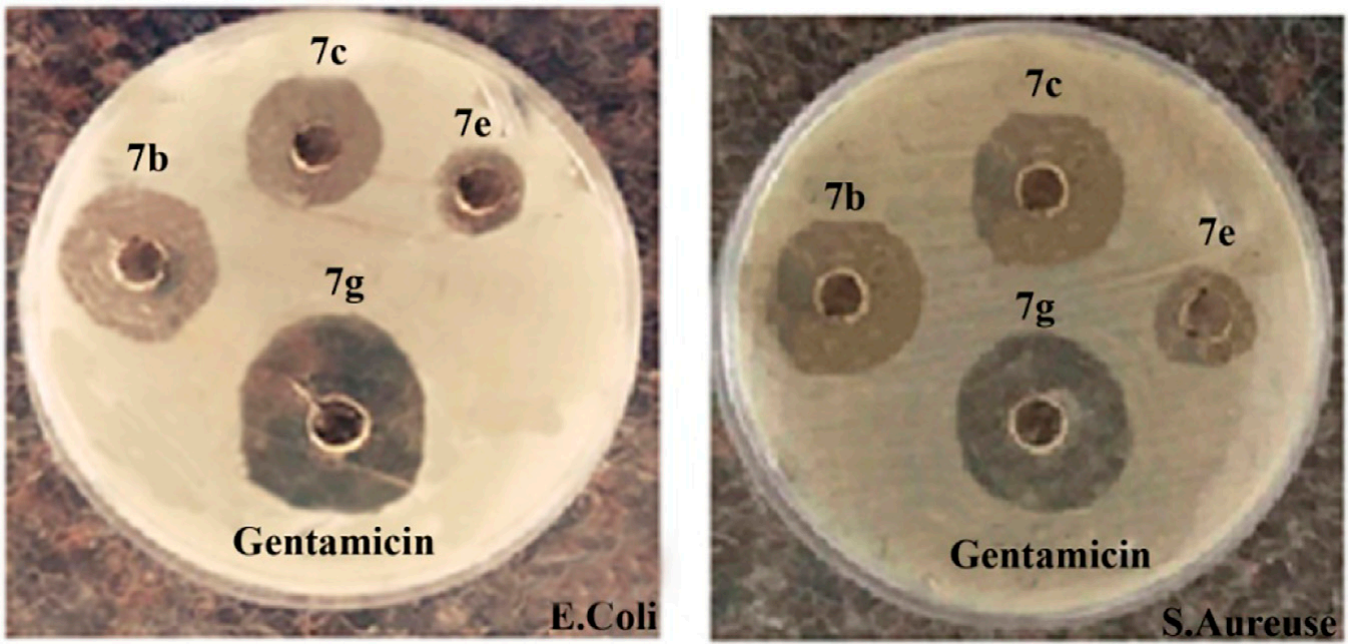
Comparison between the activity of the synthesized compounds with gentamicin.

For the investigation of the antibacterial activity of synthesized compounds, two antibiotic drugs such as streptomycin and gentamicin were employed, and the results of the antimicrobial activity of synthesized compounds compared with two standards are displayed in Table 4 and Figure 8. For the evaluation of this experiment, two suitable and significant factors that effect the diameter and inhibition zone are the type of bacteria and concentration of synthesized spiropyridoindolepyrrolidines. Spiropyridoindolepyrrolidines **7a-7g** have good diameter and inhibition zone owing to the effect of *Escherichia coli*, among the Gram-positive and -negative bacteria (Figure 8).

**TABLE 4:** Antibacterial activity of spiropyridoindolepyrrolidines **7a-7g**.

*Compound*	*Staphylococcus aureus* (+)	*Bacillus cereus* (+)	*Escherichia coli* (-)	*Klebsiella pneumoniae* (-)
**7a**	9	11	10	8
**7b**	**18**	**21**	**22**	**19**
**7c**	**17**	**21**	**22**	**18**
**7d**	10	7	11	8
**7e**	**17**	**19**	**20**	**22**
**7f**	10	9	11	12
**7g**	**19**	**23**	**24**	**22**
**Streptomycin**	18	24	25	23
**Gentamicin**	21	23	24	21

Bold indicates best condition for performing reactions.

## 3 Molecular geometry

**FIGURE 9:**
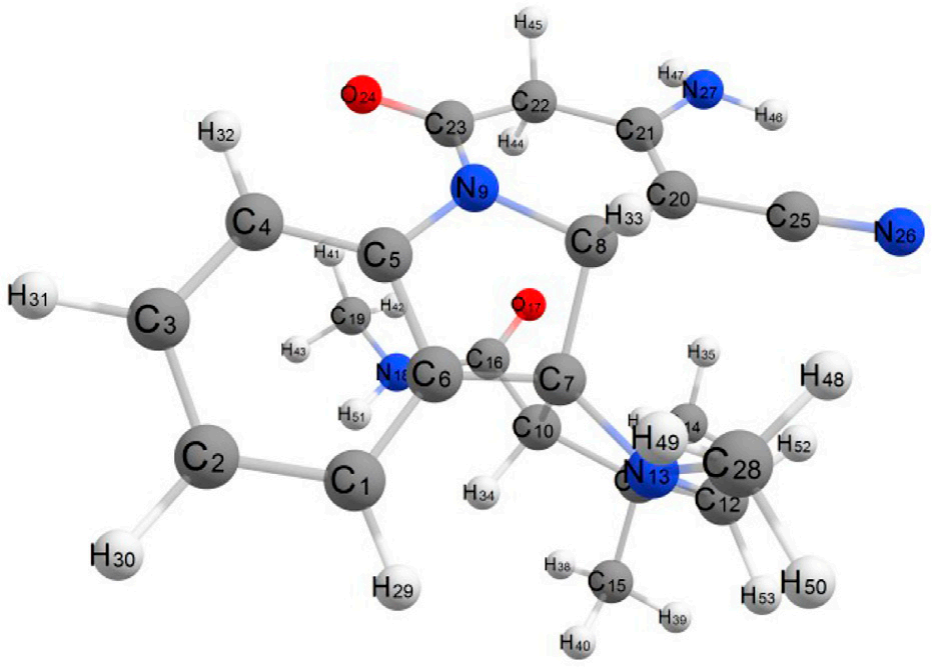
Optimized molecular structure.

An optimized structure with a numbering scheme is shown in Figure 9. For the four compounds studied, the bond lengths of C-H bonds are calculated as 1.08–1.10 Å. The C-N bond length for the four states in the range of 1.36–1.45 Å C25-N26 bond length is considerably shorter than other C-N bond lengths because of the hybridization of sp. The C16 = O17, C23 = O24 bond lengths are calculated as 1.22–1.23 Å. The sp^3^ hybridized carbon atom bonded with C22 (sp^2^)—C21 (sp^3^) atoms shows their bond lengths 1.49 Å and 1.50 Å, respectively. The bond length of C20 (sp^2^)—C21 (sp^2^) is 1.36 Å, which is slightly lower and shows its double bond character.

### Mulliken atomic charges

**FIGURE 10:**
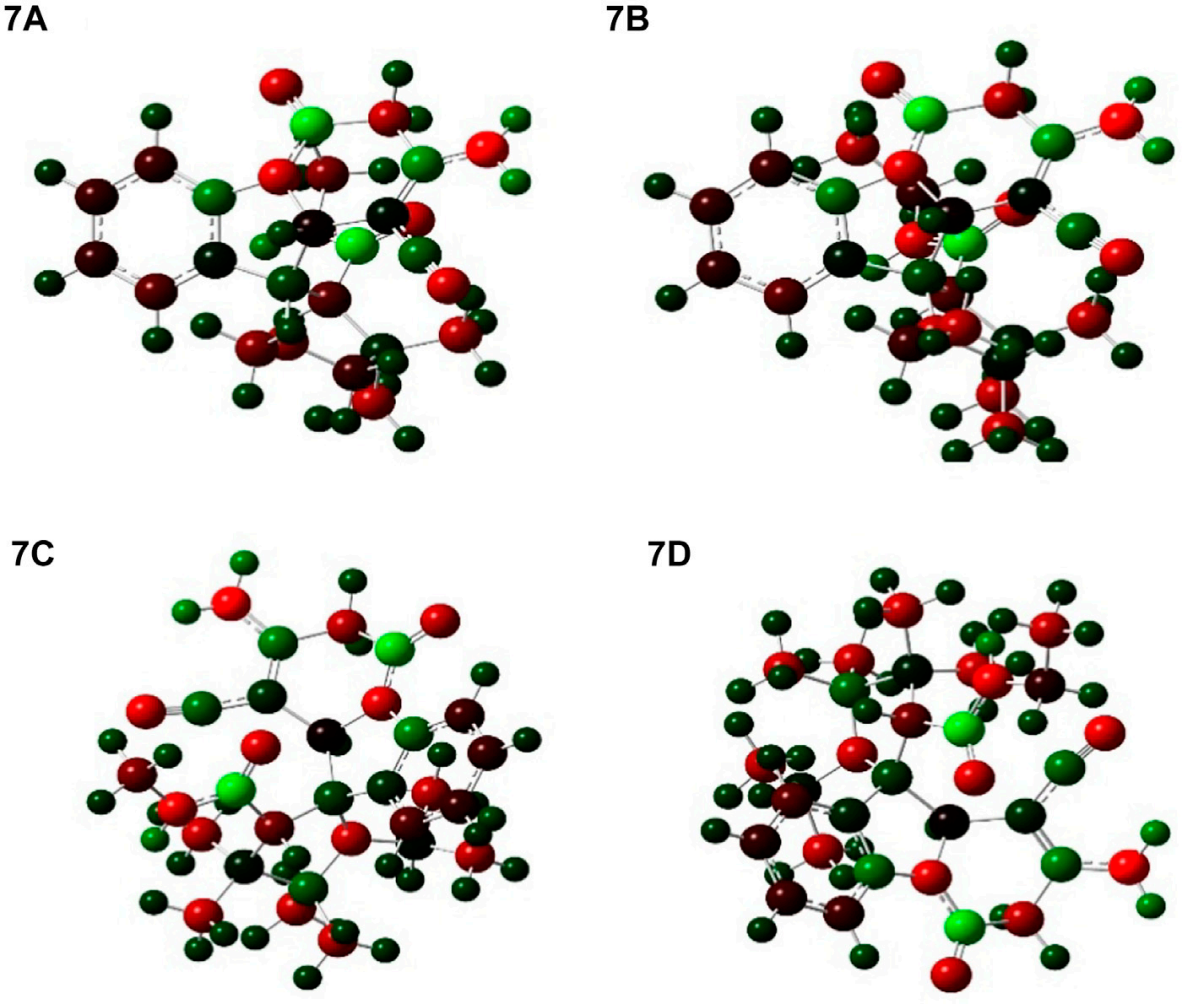
Distribution of Mulliken atomic charge for **7a-7d**.

The atomic charges were calculated by Mulliken atomic charges (Mulliken, 1955). The electronic charge on atoms within a molecule is a crucial factor to decide the bonding capability of the molecule. The net atomic charge in the molecule provides a picture of electron density distribution over the molecule. The distribution of Mulliken atomic charge is listed in Table 5 and shown in Figure 10. The Mulliken atomic charges on carbon atoms reveal either the positive or negative value. All hydrogen atoms showed a net positive charge, but H_46,_ H_47,_ and H_51_ obtained high value positive charge than the other hydrogen atoms, due to the presence of an electronegative atom (N). All oxygen atoms of the optimized compound were shown to have a negative charge, which act as donor atoms. The results are reported in Table 5 of atoms attached to a high electronegative atom.

**TABLE 5:** Mulliken atomic charges.

	Mulliken			
	7a	7b	7c	7d
**C5**	0.324458	0.327437	0.323153	0.320172
**C8**	−0.050852	−0.066118	−0.046142	−0.036001
**C16**	0.630508	0.665529	0.640289	0.639226
**C19**	−0.319524	−0.092239	−0.308651	−0.114158
**C20**	0.051492	0.133949	0.126907	0.121522
**C21**	0.388736	0.355951	0.321644	0.323730
**C23**	0.613744	0.596007	0.589181	0.592075

### *HOMO–LUMO* analysis and global reactivity descriptors

**FIGURE 11:**
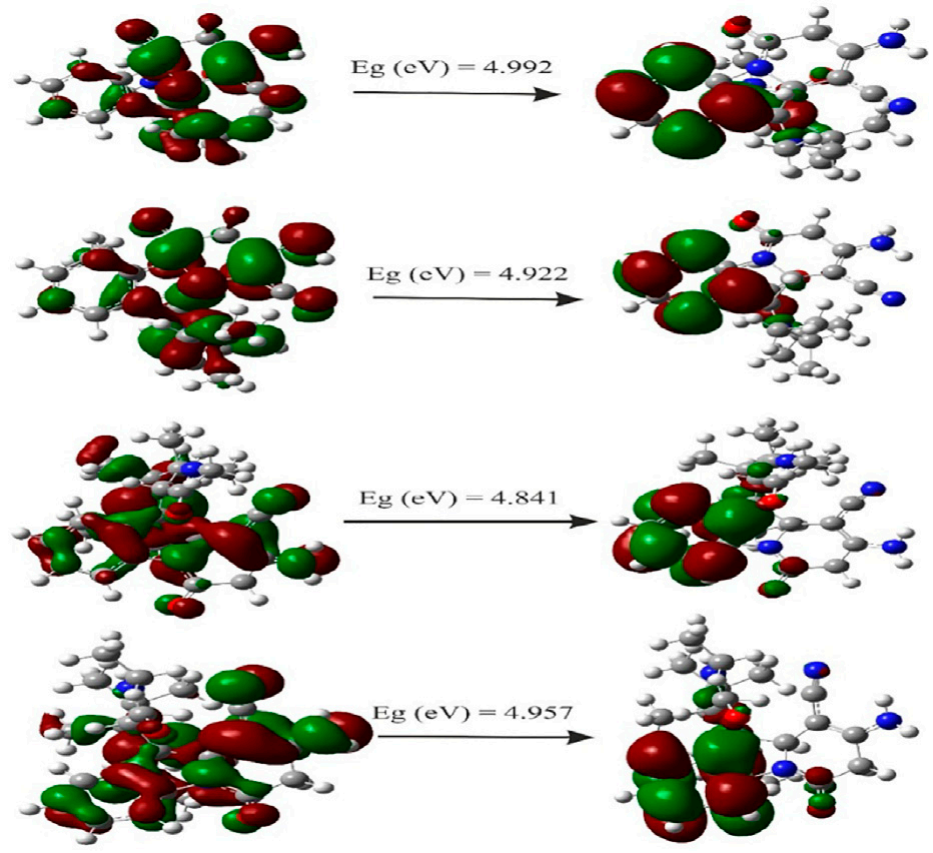
Plot HOMO and LUMO orbitals for 7a–7d compounds.

HOMO and LUMO are important parameters used in quantum chemistry. The difference between the energies of HOMO and LUMO is referred as the energy gap, which determines the reactivity and kinetic stability of molecules (Venkatesh et al., 2016). The HOMO–LUMO energy gap value for title compounds is given in Table 6. The pictorial illustration of the frontier molecular orbitals with their energies is shown in Figure 11. Furthermore, HOMO orbitals are mostly localized on Spiro and phenyl rings, while LUMO orbitals were located partially on carbonyl, but mostly located near the phenyl ring.

**TABLE 6:** Energies, band gap, dipole moment (Debye), and electronegativity.

Compounds	Energy (eV)	E_LUMO_ (eV)	E_HOMO_ (eV)	Eg (eV)	Gap (Ev)	D (Debye)	χ
**7a**	-33728.55	-0.01926	-0.20271	0.18345	4.992	2.75	3.02
**7b**	-36937.873	-0.01656	-0.19743	0.18087	4.922	2.97	2.91
**7c**	-38006.657	-0.02117	-0.19908	0.17791	4.841	3.86	3.00
**7d**	-39076.357	-0.02077	-0.20295	0.18218	4.957	3.53	3.04

Density functional theory-based global chemical reactivity descriptors such as the chemical potential (µ), chemical hardness (η), global softness (S), and electrophilicity index (ω) have been calculated for four compounds, given in Table 7. The values of chemical potential (µ) indicated that 7b compound is higher than 7a, 7c, and 7d compounds; it means that 6d compounds increase reactivity. The hardness of 7a and 7c compounds decreased, but for 7b and 7d, the chemical hardness increased. The electrophilicity by 7c and 7d compounds increases, but for 7a and 7b compounds, the electrophilicity decreased. Global chemical reactivity descriptors are used to understand the relationship between structure, stability, and global chemical reactivity of compounds. The small values of η are said to be chemically soft (S) and are highly polarizable and more chemically reactive. The chemical potential (μ) measures the escaping tendency of electrons from a compound in its ground state. High values of chemical potential signify that the molecule is less stable and more reactive (Mebi, 2011). The global electrophilicity index (ω) measures the stabilization in energy of a system, when it acquires an additional electronic charge from the environment. The results of the four compounds studied revealed that 6b has high reactivity, and the resultant antioxidant ability showed good activity that **7b > 7c > 7a>7d.**

**TABLE 7:** Molecular descriptor results.

Compound	μ	η	S	ω	I	A
**7a**	−3.020	2.496	0.401	7.309	4.99	5.52
**7b**	−2.912	2.461	0.406	6.889	4.92	5.37
**7c**	−2.997	2.421	0.413	7.420	4.84	5.42
**7d**	−3.044	2.479	0.403	7.476	4.96	5.52

### Molecular electrostatic potential surface

**FIGURE 12:**
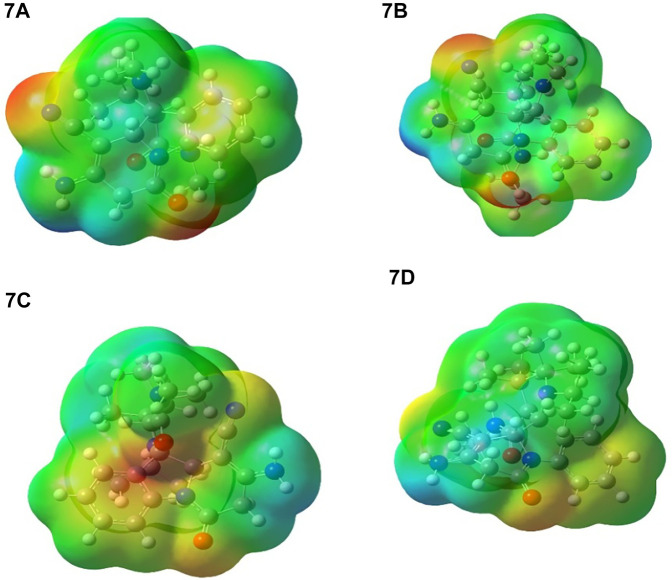
Calculated molecular electrostatic potential surfaces for **7a-7d.**

The molecular electrostatic potential (MEP) is used to predict the relative reactivity positions in a species for nucleophilic and electrophilic attack. The electrostatic potential surface map of the studied compound is given in Figure 12. The color of the compounds is in the range of −0.144e^−2^ to +0.144e^−2^. The red and blue color in the MEP structure point to more electron-rich and electron-poor region, respectively. In the MEP, the negative potential regions are localized over the electronegative atoms (oxygen and nitrogen) and the positive potential regions are localized over the carbon and hydrogen atoms and phenyl ring. However, the nitrogen atom of the compound is a less-negative potential site than the other electronegative atoms. Therefore, the more-negative potential and positive electrostatic potential sites are more favorable for the attraction of nucleophilic and electrophilic species.

### Vibrational assignments

**FIGURE 13:**
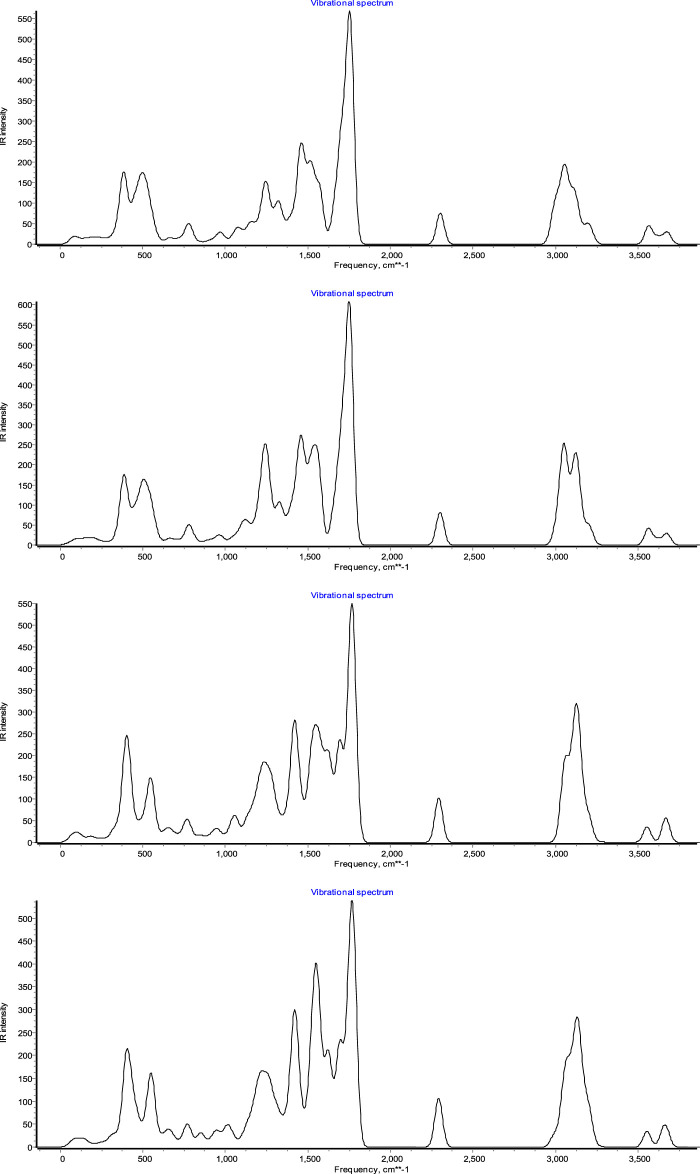
Plot vibration spectrum for **7a-7d** compounds.

There are a total of 53–68 atoms in **7a-7d** compounds with 153–198 fundamental modes of vibration. The experimental FTIR spectrum was obtained from this article. Theoretically, frequencies are predicted by the DFT/B3LYP method using the 6–311++G** basis set. The theoretically predicted vibrational frequencies are scaled by an empirical factor 0.9613 (Foresman and Frisch, 1996). The infrared stretching frequencies of these groups vary in the same order, ranging from 1,222–1,254 cm^−1^ for C-N and 2,210 cm^−1^ for C=N (cyanide). The experimental C=O stretching frequency appeared at 1767–1779 cm^−1^, and theoretically, it is observed at 1,684–1,687 cm^−1^. Computed frequencies at 3,000–3,264 cm^−1^ indicate C-H stretching. The aromatic C=C stretching frequency theoretically appeared at 1,664 cm^−1^ and 1,651–1,656 for C=C aromatic, which is close to the experimental values. Also, frequencies at 3,421 and 3,528 cm^−1^ for NH stretching in Figure 13 show all vibration spectra.

## 4 Conclusion

In summary, we have investigated an effective, green, and environment-friendly reaction including isatins, activated acetylenic compounds, alkyl bromides, ammonium acetate, isothiocyanates, and aziridine in aqueous media at ambient temperature in the presence of a new organometallic nanocatalyst Ag/Fe_3_O_4_/CdO@MWCNT-MNC, which has produced new derivatives of spiropyridoindolepyrrolidines. Also, two methods were employed for the evaluation of the antioxidant power of the synthesized spiropyridoindolepyrrolidines **7a-7d** which confirmed that the synthesized compounds have good antioxidant ability relative to standard antioxidants. In addition, for the investigation of the antibacterial activity of synthesized compounds, Gram-positive and -negative bacteria were utilized, and the disk diffusion process was used for the confirmation of the antimicrobial ability of the produced spiropyridoindolepyrrolidines. The results of antimicrobial activity investigation displays that synthesized spiropyridoindolepyrrolidines have good biological activity and could avoid the bacterial growth. As a result, this procedure that is used for the synthesis of spiropyridoindolepyrrolidines have some advantages such as reaction with high rate, high yield product, green process, employing little amounts of catalyst, easy separation of organometallic catalyst from the reaction mixture, and easy product purification that are the important points in these reactions.

## 5 Experimental

### General

In this research work, all the starting materials that are needed for the synthesis of spiropyridoindolepyrrolidines and also reagents and solvents were prepared from Fluka and Merck Company without any purification. For the synthesis of the nanocatalyst, MWCNTs that have 8 nm diameter are used, of 30 μm for length and 95% for purity, and prepared from Merck Company. For approving the construction of synthesized catalyst Ag/Fe_3_O_4_/CdO@MWCNT MNCs, the spectroscopy analysis such as XRD, SEM, EDX, and VSM was utilized. The FT-IR (KBr medium) of synthesized spiropyridoindolepyrrolidines was taken by using the Shimadzu IR-460 spectrometer instrument. Additionally, another way for the confirmation of the structure of synthesized compounds giving ^1^H-NMR and ^13^C-NMR with the Bruker DRX-500 AVANCE spectrometer instrument with 500 MHz NMR in CDCl_3_ as solvent and TMS as internal standard was used. Mass spectra for synthesized compounds were given by the Finnigan, MAT 8430 spectrometer with an ionization potential 70 eV. For the determination of elements in the prepared compounds, the Heraeus CHN–O-Rapid analyzer was employed.

### Generation of *Petasites hybridus* rhizome water extract

*Petasites hybridus* rhizome was dried, and 10 g of it was poured in a two-neck round-bottom flask (250 ml), and water (100 ml) was added, and the new mixture was stirred at 100^o^C and filtered after 2 h. For the production of the nanocomposite, the water extract of *Petasites hybridus* rhizome and other compounds was employed as follows.

### Generation of Ag/Fe_3_O_4_/CdO@MWCNT MNCs (Rajendran and Sengodan, 2017)

The mixture of Cd (NO_3_)_2_ (1.5 g) and FeCl_2_.4H_2_O (1.5 g) were dissolved in water (10 ml), and then, the *Petasites hybridus* rhizome water extract (5 ml) was added easily to the previous mixture, and the temperature of the mixture was enhanced to 100^o^C in a round-bottom flask and mixed for 5 h When the reaction was completed, the temperature of the reaction was reduced to room temperature. Then, after cooling, for deleting the undesired organic compounds, the reaction mixture was sonicated for 30 min and centrifuged at 7,000 rpm for about 10 min. After this, AgNO_3_ (1.5 g) was poured in the previous mixture, and sonication of the new mixture was continued at 100°C for 45 min, and Ag/Fe_3_O_4_/CdO MNCs was synthesized. For the synthesis of Ag/Fe_3_O_4_/CdOQMWCNTs MNCs, the MWCNTs (0.1 g) and Ag/Fe_3_O_4_//CdO MNCs (0.1 g) prepared in the previous section were added to 100 ml water extract of *Petasites hybridus* rhizome and mixed at 150^o^C for 1 h. Centrifugation was used for the separation of colloid and then washed with water, dried, and calcinated at 300°C for 45 min. After this, the Ag/Fe_3_O_4_/CdO @MWCNT magnetic nanocomposite was produced, which was cooled to room temperature and washed with a mixture of water and ethanol (50:50) several times. After washing the solid, by employing an external magnet, the catalyst was separated and dried at room temperature for 24 h.

### Preparation process of spiropyridoindolepyrrolidine 7a–l

Isatines **1** (2 mmol), acetyl chloride **2** (2 mmol), and Ag/Fe_3_O_4_/CdO@MWCNT (0.02 g) were added to water as solvent at room temperature and mixed for 30 min. Secondary amines **3** (2 mmol) were poured to the previous mixture after 30 min, and the new mixture was mixed for 20 min. Vinilidene Meldrum’s acid **4** (2 mmol) and amines **5** (2 mmol) were added to the previous mixture and stirred for 30 min. Finally, malonnnitrile **6** (2 mmol) was added, and the final mixture was stirred for 45 min in the presence of a catalyst. Completion of the reaction took place after 3 h and monitored by TLC; in this stage, separation was performed with an external magnet, and solid residue was washed by EtOH and Et_2_O to prepare purified spiropyridoindolepyrrolidine **7** (see Supplementary Material).

***8-Amino-9-cyano-N,1′,4′,4′-tetramethyl-6-oxo-6H-spiro[pyrido[1,2-a]indole-10,2′-pyrrolidine]-3′-carboxamide*** (**7a):** yellow powder, m. p. 135–137°C, 0.72 g, yield 95%. IR (KBr) (ν_max_/cm^−1^): 3,456, 1,732, 2,198, 1,698, 1,624, 1,456, and 1,230 cm^−1^.^1^H NMR (500 MHz, CDCl_3_): *δ* 1.05 (3 H, s, Me), 1.10 (3 H, s, Me), 2.39 (3 H, s, NMe), 2.76 (3 H, s, NMe), 2.82 (1 H, d, ^2^ *J*_HH_ = 8.0 Hz, CH), 3.04 (1 H, d, ^2^ *J*_HH_ = 8.0 Hz, CH), 3.64 (1 H, s, CH), 5.68 (1 H, s, CH), 7.08 (1 H, t, ^3^ *J*_HH_ = 7.6 Hz, CH), 7.20 (1 H, d, ^3^ *J*_HH_ = 8.0 Hz, CH), 7.39 (1 H, t, ^3^ *J* = 7.6 Hz, CH), 7.51 (1 H, d, ^3^ *J* = 7.6 Hz, CH), 9.87 (1 H, s, NH), and 10.23 (2 H, s, NH_2_) ppm. ^13^C NMR (125.7 MHz, CDCl_3_): *δ* 25.56, 25.95, 38.06, 40.29, 63.08, 65.10, 70.20, 79.57, 95.19, 115.18, 117.01, 123.20, 124.24, 128.55, 133.86, 136.93, 154.40, 160.91, 160.92, and 171.97 ppm. MS, *m/z* (%): 377 (M^+^, 10), 319 (48), and 58 (100). Anal. Calcd for C_21_H_23_N_5_O_2_ (377.45): C, 66.83; H, 6.14; N, 18.55; Found: C, 66.92; H, 6.23; N, 18.74%.

***8-Amino-9-cyano-N,1′-diethyl-4′,4′,5′-trimethyl-6-oxo-6H-spiro[pyrido[1,2-a]indole-10,2′-pyrrolidine]-3′-carboxamide* (7b):** yellow powder, m. p. 142–144°C, 0.76 g, yield 90%. IR (KBr) (ν_max_/cm^−1^): 3,457, 1,732, 2,196, 1,697, 1,626, 1,585, and 1,227 cm^−1^.^1^H NMR (500 MHz, CDCl_3_): *δ* 1.02 (3 H, s, Me), 1.07 (3 H, s, Me), 1.10 (3 H, d, ^3^ *J*_HH_ = 8.0 Hz, Me), 1.19 (3 H, t, ^3^ *J*_HH_ = 7.4 Hz, Me),1.22 (3 H, t, ^3^ *J*_HH_ = 7.4 Hz, Me), 2.81–2.86 (1 H, m, CH), 2.96–3.01 (1 H, m, CH), 3.20–3.27 (3 H, m, 3 CH), 3.67 (1 H, s, CH), 5.68 (1 H, s, CH), 7.08 (1 H, t, ^3^ *J*_HH_ = 7.6 Hz, CH), 7.19 (1 H, d, ^3^ *J*_HH_ = 7.6 Hz, CH), 7.39 (1 H, t, ^3^ *J* = 7.6 Hz, CH), 7.52 (1 H, d, ^3^ *J* = 7.6 Hz, CH), 10.04 (1 H, s, NH), and 10.37 (2 H, s, NH_2_) ppm. ^13^C NMR (125.7 MHz, CDCl_3_): *δ* 14.61, 14.70, 15.44, 23.76, 35.51, 41.32, 42.38, 61.91, 65.14, 70.19, 77.47, 95.19, 115.18, 117.01, 123.17, 124.19, 128.65, 133.90, 136.72, 154.51, 160.91, 161.83, and 171.24 ppm. MS, *m/z* (%): 419 (M^+^, 15), 347 (38), and 72 (100). Anal. Calcd for C_24_H_29_N_5_O_2_ (419.53): C, 68.71; H, 6.97; N, 16.69; Found: C, 68.83; H, 7.06; N, 16.83%.

***8-Amino-9-cyano-1′-isopropyl-N,4′,4′,5′,5′-pentamethyl-6-oxo-6H-spiro[pyrido[1,2-a]indole-10,2′-pyrrolidine]-3′-carboxamide*** (**7c**)**:** yellow powder, m. p. 152–154°C, 0.78 g, yield 90%. IR (KBr) (ν_max_/cm^−1^): 3,458, 2,217, 1719, 1,694, 1,628, 1,597 and 1,257 cm^−1^.^1^H NMR (500 MHz, CDCl_3_): *δ* 1.08 (3 H, s, Me), 1.13 (3 H, s, Me), 1.16 (6 H, d, ^3^ *J*_HH_ = 7.2 Hz, 2 Me), 1.22 (3 H, s, Me), 1.27 (3 H, s, Me), 2.76 (3 H, s, NMe), 3.57–3.61 (1 H, m, CH), 3.71 (1 H, s, CH), 5.67 (1 H, s, CH), 7.06 (1 H, t, ^3^ *J*_HH_ = 7.6 Hz, CH), 7.21 (1 H, d, ^3^ *J*_HH_ = 7.6 Hz, CH), 7.42 (1 H, t, ^3^ *J* = 7.6 Hz, CH), 7.52 (1 H, d, ^3^ *J* = 7.6 Hz, CH), 10.07 (1 H, s, NH), 10.42 (2 H, s, NH_2_) ppm.^13^C NMR (125.7 MHz, CDCl_3_): *δ* 22.51, 23.39, 23.99, 25.95, 46.83, 47.38, 55.30, 67.80, 70.12, 78.33, 95.19, 115.19, 117.01, 123.34, 124.15, 128.74, 134.33, 136.96, 154.51, 160.91, 161.65, 172.50 ppm. MS, *m/z* (%): 433 (M^+^, 15), 375 (48), 58 (100). Anal. Calcd for C_25_H_31_N_5_O_2_ (433.56): C, 69.26; H, 7.21; N, 16.15; Found: C, 69.38; H, 7.32; N, 16.26%.

***8-Amino-9-cyano-N-ethyl-1′-isopropyl-4′,4′,5′,5′-tetramethyl-6-oxo-6H-spiro [pyrido[1,2-a]indole-10,2′-pyrrolidine]-3′-carboxamide*** (**7d**)**:** yellow powder, m. p. 163–165°C, 0.78 g, yield 87%. IR (KBr) (ν_max_/cm^−1^): 3,464, 2,184, 1,719, 1,694, 1,628, 1,597, and 1,257 cm^−1^.^1^H NMR (500 MHz, CDCl_3_): *δ* 1.08 (3 H, s, Me), 1.13 (3 H, s, Me), 1.17 (6 H, d, ^3^ *J*_HH_ = 6.8 Hz, 2 Me), 1.22 (3 H, s, Me), 1.23 (3 H, t, ^3^ *J*_HH_ = 7.4 Hz, Me), 1.27 (3 H, s, Me), 3.21–3.27 (2 H, m, CH_2_N), 3.58–3.61 (1 H, m, CH), 3.75 (1 H, s, CH), 5.68 (1 H, s, CH), 7.08 (1 H, t, ^3^ *J*_HH_ = 7.7 Hz, CH), 7.22 (1 H, d, ^3^ *J*_HH_ = 7.7 Hz, CH), 7.42 (1 H, t, ^3^ *J* = 7.7 Hz, CH), 7.53 (1 H, d, ^3^ *J* = 7.6 Hz, CH), 9.87 (1 H, s, NH), and 10.23 (2 H, s, NH_2_) ppm. ^13^C NMR (125.7 MHz, CDCl_3_): δ 14.70, 22.51, 23.39, 23.99, 35.51, 46.67, 47.38, 54.94, 67.80, 70.12, 78.46, 95.19, 115.19, 117.01, 123.34, 124.15, 128.74, 134.33, 136.96, 154.51, 160.91, 161.65, and 172.04 ppm. MS, *m/z* (%): 447 (M^+^, 15), 375 (48), and 72 (100). Anal. Calcd for C_26_H_33_N_5_O_2_ (447.58): C, 69.77; H, 7.43; N, 15.65; Found: C, 69.83; H, 7.52; N, 15.73%.

***8-Amino-9-cyano-N-ethyl-1′,4′-dimethyl-6-oxo-6H-spiro[pyrido[1,2-a]indole-10,2′-pyrrolidine]-3′-carboxamide*** (**7e**)**:** yellow powder, m. p. 151–153°C, 0.66 g, yield 87%. IR (KBr) (ν_max_/cm^−1^): 3,468, 2,196, 1,716, 1,696, 1,663, 1,547, 1,473, and 1,228 cm^−1^.^1^H NMR (500 MHz, CDCl_3_): *δ* 1.07 (3 H, d, ^3^ *J*_HH_ = 8.0 Hz, Me), 1.23 (3 H, t, ^3^ *J*_HH_ = 7.4 Hz, Me), 2.30–2.35 (1 H, m, CH), 2.44 (NCH_3_), 3.02 (2 H, m, CH_2_), 3.22 (2 H, q, ^3^ *J*_HH_ = 7.4 Hz, CH_2_N), 3.55–3.57 (1 H, m, CH), 5.65 (1 H, s, CH), 7.06 (1 H, t, ^3^ *J*_HH_ = 7.7 Hz, CH), 7.22 (1 H, d, ^3^ *J*_HH_ = 7.6 Hz, CH), 7.40 (1 H, t, ^3^ *J* = 7.6 Hz, CH), 7.52 (1 H, d, ^3^ *J* = 7.6 Hz, CH), 10.12 (1 H, s, NH), and 10.56 (2 H, s, NH_2_) ppm. ^13^C NMR (125.7 MHz, CDCl_3_): δ 14.70, 16.52, 32.15, 35.61, 39.39, 54.34, 59.46, 70.32, 79.07, 95.22, 115.17, 117.01, 123.41, 124.18, 128.54, 133.12, 137.28, 154.40, 160.91, 161.54, and 174.46 ppm. MS, *m/z* (%): 377 (M^+^, 10), 375 (68), and 72 (100). Anal. Calcd for C_21_H_23_N_5_O_2_ (377.45): C, 66.83; H, 6.14; N, 18.55; Found: C, 66.92; H, 6.34; N, 18.68%.

***8-Amino-9-cyano-N,1′,4′-trimethyl-6-oxo-6H-spiro[pyrido[1,2-a]indole-10,2′-pyrrolidine]-3′-carboxamide*** (**7f**)**:** yellow powder, m. p. 147–149°C, 0.64 g, yield 87%. IR (KBr) (ν_max_/cm^−1^): 3,457, 2,213, 1,716, 1,697, 1,663, 1,547, 1,473, and 1,228 cm^−1^.^1^H NMR (500 MHz, CDCl_3_): *δ* 1.07 (3 H, d, ^3^ *J*_HH_ = 8.0 Hz, Me), 2.31–2.36 (1 H, m, CH), 2.45 (NCH_3_), 2.76 (NCH_3_), 3.02 (2 H, m, CH_2_), 3.58–3.60 (1 H, m, CH), 5.68 (1 H, s, CH), 7.06 (1 H, t, ^3^ *J*_HH_ = 7.7 Hz, CH), 7.23 (1 H, t, ^3^ *J*_HH_ = 7.6 Hz, CH), 7.38 (1 H, d, ^3^ *J* = 7.6 Hz, CH), 7.52 (1 H, d, ^3^ *J* = 7.6 Hz, CH), 10.15 (1 H, s, NH), and 10.63 (2 H, s, NH_2_) ppm.^13^C NMR (125.7 MHz, CDCl_3_): δ 16.52, 25.98, 32.10, 39.39, 53.73, 59.46, 70.32, 79.07, 95.22, 115.17, 117.01, 123.41, 124.18, 128.54, 133.12, 137.28, 154.40, 160.91, 161.54, and 174.96 ppm. MS, *m/z* (%): 363 (M^+^, 10), 375 (68), and 45 (100). Anal. Calcd for C_20_H_21_N_5_O_2_ (363.42): C, 66.10; H, 5.82; N, 19.27; Found: C, 66.25; H, 5.93; N, 19.34%.

***8-Amino-9-cyano-N,1′-dimethyl-6-oxo-4′-phenyl-6H-spiro[pyrido[1,2-a]indole-10,2′-pyrrolidine]-3′-carboxamide*** (**7g):** yellow powder, m. p. 171–173°C, 0.77 g, yield 90%. IR (KBr) (ν_max_/cm^−1^): 3,387, 2,198, 1,710, 1,695, 1,635, 1,460, 1,213, and 1,092 cm^−1^.^1^H NMR (500 MHz, CDCl_3_): *δ* 2.44 (3 H, s, NMe), 2.75 (3 H, s, NMe), 3.15–3.18 (1 H, m, CH), 3.41–3.45 (1 H, m, CH), 3.51–3.53 (1 H, m, CH), 3.69 (1 H, d, ^3^ *J* = 6.7 Hz, CH), 5.68 (1 H, s, CH), 7.07 (1 H, t, ^3^ *J*_HH_ = 7.8 Hz, CH), 7.22–7.29 (6 H, m, 6 CH), 7.40 (1 H, t, ^3^ *J* = 7.8 Hz, CH), 7.52 (1 H, d, ^3^ *J* = 7.8 Hz, CH), 10.15 (1 H, s, NH), and 10.38 (2 H, s, NH_2_) ppm. ^13^C NMR (125.7 MHz, CDCl_3_): δ 25.96, 39.43, 44.49, 55.58, 56.88, 70.32, 78.05, 95.19, 115.17, 117.01, 123.43, 124.14, 126.20, 126.81, 128.54, 128.60, 132.73, 137.13, 139.91, 154.50, 160.91, 161.46, and 174.52 ppm. MS, *m/z* (%): 425 (M^+^, 10), 77 (54), and 58 (100). Anal. Calcd for C_25_H_23_N_5_O_2_ (425.49): C, 70.57; H, 5.45; N, 16.46; Found: C, 70.68; H, 5.56; N, 16.57%.

***8-Amino-9-cyano-N-(4-methoxybenzyl)-1′,4′-dimethyl-6-oxo-6H-spiro[pyrido[1,2-a]indole-10,2′-pyrrolidine]-3′-carboxamide*** (**7h**)**:** yellow powder, m. p. 167–169°C, 0.80 g, yield 85%. IR (KBr) (ν_max_/cm^−1^): 3,689, 2,132, 1,716, 1,697.3, 1,663, 1,547, 1,473, and 1,228 cm^−1^.^1^H NMR (500 MHz, CDCl_3_): *δ* 1.07 (3 H, d, ^3^ *J*_HH_ = 8.0 Hz, Me), 2.30–2.35 (1 H, m, CH), 2.44 (3 H, s, NMe), 3.02 (2 H, m, CH_2_), 3.60–3.62 (1 H, m, CH), 3.78 (3 H, s, MeO), 4.42 (2 H, s, CH_2_), 5.69 (1 H, s, CH), 6.85 (2 H, d, ^3^ *J* = 7.8 Hz, 2 CH), 7.06 (1 H, t, ^3^ *J* = 7.6 Hz, CH), 7.18 (2 H, d, ^3^ *J*_HH_ = 7.8 Hz, 2 CH), 7.23 (1 H, d, ^3^ *J*_HH_ = 7.6 Hz, CH), 7.38 (1 H, t, ^3^ *J* = 7.6 Hz, CH), 7.52 (1 H, d, ^3^ *J* = 7.6 Hz, CH), 9.86 (1 H, s, NH), and 10.37 (2 H, s, NH_2_) ppm. ^13^C NMR (125.7 MHz, CDCl_3_): *δ* 16.52, 32.15, 39.39, 44.15, 54.61, 55.33, 59.46, 70.32, 79.10, 95.22, 113.79, 115.17, 117.01, 123.41, 124.18, 128.54, 128.78, 133.13, 133.98, 137.28, 158.76, 154.40, 160.91, 161.54, and 173.66 ppm. MS, *m/z* (%): 495 (M^+^, 10), 375 (54), and 121 (100). Anal. Calcd for C_27_H_27_N_5_O_3_ (469.55): C, 69.07; H, 5.80; N, 14.92; Found: C, 68.82; H, 6.14; N, 11.97%.

***8-Amino-9-cyano-N,4′,4′,5′-tetramethyl-1'-(4-nitrophenyl)-6-oxo-6H-spiro[pyrido [1,2-a]indole-10,2′-pyrrolidine]-3′-carboxamide*** (**7i**)**:** orange powder, m. p. 183–185°C, 0.72 g, yield 75%. IR (KBr) (ν_max_/cm^−1^): 3,468, 2,198, 1,710, 1,698, 1,628, 1,439, and 1,219 cm^−1^.^1^H NMR (500 MHz, CDCl_3_): *δ* 1.03 (3 H, s, Me), 1.08 (3 H, s, Me), 1.22 (3 H, d, ^3^ *J* = 6.8 Hz, Me), 2.75 (3 H, s, NMe), 3.74 (1 H, s, CH), 4.06 (1 H, q, ^3^ *J* = 6.8 Hz, CH), 5.71 (1 H, s, CH), 6.98 (2 H, d, ^3^ *J* = 7.8 Hz, 2 CH), 7.10 (1 H, t, ^3^ *J* = 7.6 Hz, CH), 7.23 (1 H, d, ^3^ *J*_HH_ = 7.6 Hz, CH), 7.42 (1 H, t, ^3^ *J*_HH_ = 7.6 Hz, CH), 7.52 (1 H, d, ^3^ *J* = 7.6 Hz, CH), 8.08 (2 H, d, ^3^ *J* = 7.6 Hz, 2 CH), 10.03 (1 H, s, NH), and 10.28 (2 H, s, NH_2_) ppm. ^13^C NMR (125.7 MHz, CDCl_3_): *δ* 14.88, 24.10, 25.95, 44.98, 63.34, 64.74, 69.91, 78.36, 95.58, 115.19, 116.27, 117.02, 124.21, 124.59, 125.34, 128.77, 133.16, 136.70, 144.43, 151.03, 154.13, 161.44, 163.21, and 171.36 ppm. MS, *m/z* (%): 498 (M^+^, 15), 440 (52), and 58 (100). Anal. Calcd for C_27_H_26_N_6_O_4_ (498.5452): C, 65.05; H, 5.26; N, 16.86; Found: C, 65.18; H, 5.38; N, 16.97%.

***8-Amino-9-cyano-N,4′,4′-trimethyl-6-oxo-1'-(p-tolyl)-6H-spiro[pyrido[1,2-a]indole-10,2′-pyrrolidine]-3′-carboxamide*** (**7j**)**:** pale orange powder, m. p. 177–179°C, 0.73 g, yield 80%. IR (KBr) (ν_max_/cm^−1^): 3,437, 2,214, 1,744, 1,697, 1,634, 1,573, 1,439, and 1,215 cm^−1^.^1^H NMR (500 MHz, CDCl_3_): *δ* 1.08 (3 H, d, ^3^ *J* = 6.8 Hz, Me), 2.28 (3 H, s, Me), 2.31–2.34 (1 H, m, CH), 2.35 (3 H, s, Me), 2.75 (3 H, s, NMe), 3.66–3.79 (3 H, m, 3 CH), 5.71 (1 H, s, CH), 6.84 (2 H, d, ^3^ *J*_HH_ = 7.8 Hz, 2 CH), 7.03 (2 H, d, ^3^ *J*_HH_ = 7.8 Hz, 2 CH), 7.09 (1 H, s, CH), 7.19 (1 H, d, ^3^ *J*_HH_ = 7.6 Hz, CH), 7.36 (1 H, d, ^3^ *J* = 7.6 Hz, CH), 9.85 (1 H, s, NH), and 10.67 (2 H, s, NH_2_) ppm. ^13^C NMR (125.7 MHz, CDCl_3_): *δ* 20.71, 25.50, 25.95, 43.13, 58.77, 65.98, 69.89, 79.02, 95.58, 115.18, 116.26, 117.02, 124.10, 124.22, 128.76, 129.33, 130.82, 133.18, 136.94, 144.69, 154.13, 161.44, 161.87, and 171.79 ppm. MS, *m/z* (%): 453 (M^+^, 10), 395 (68), and 58 (100). Anal. Calcd for C_27_H_27_N_5_O_2_ (453.55): C, 71.50; H, 6.00; N, 15.44; Found: C, 71.63; H, 6.18; N, 15.52%.

***8-Amino-9-cyano-4′-ethyl-N,1′,2,4′-tetramethyl-6-oxo-6H-spiro[pyrido[1,2-a]indole-10,2′-pyrrolidine]-3′-carboxamide*** (**7k**)**:** yellow powder, m. p. 1201–203°C, yield 75%. IR (KBr) (ν_max_/cm^−1^): 3,547, 3,387, 1735, 1,698, 1,678, 1,576, 1,487 and 1,295 cm^−1^.^1^H NMR (500 MHz, CDCl_3_): *δ* 0.82 (3 H, t, ^3^ *J*_HH_ = 7.4 Hz, Me), 1.05 (3 H, s, Me), 1.47–1.49 (1 H, m, CH), 1.58–1.63 (1 H, m, CH), 2.28 (3 H, s, Me), 2.37 (3 H, s, NMe), 2.76 (3 H, s, NMe), 2.78 (1 H, d, ^2^ *J* = 6.3 Hz, CH), 3.00 (1 H, d, ^2^ *J* = 6.3 Hz, CH), 3.66 (1 H, s, CH), 5.68 (1 H, s, CH), 7.07 (1 H, s, CH), 7.18 (1 H, d, ^3^ *J*_HH_ = 7.8 Hz, CH), 7.31 (1 H, d, ^3^ *J*_HH_ = 7.8 Hz, CH), 10.23 (1 H, s, NH), 10.64 (2 H, s, NH_2_) ppm. ^13^C NMR (125.7 MHz, CDCl_3_): *δ* 8.55, 21.28, 22.21, 25.95, 30.82, 38.05, 41.04, 54.32, 62.76, 70.20, 77.80, 95.19, 114.19, 117.01, 123.59, 128.84, 134.48, 135.24, 135.64, 154.50, 160.91, 160.93, 172.05 ppm. MS, *m/z* (%): 405 (M^+^, 10), 395 (68), 58 (100). Anal. Calcd for C_23_H_27_N_5_O_2_ (405.50): C, 68.13; H, 6.71; N, 17.27; Found: C, 68.32; H, 6.34; N, 17.42%.

***8-Amino-9-cyano-1′,2,4′,4′-tetramethyl-6-oxo-N-phenyl-6H-spiro[pyrido[1,2-a]indole-10,2′-pyrrolidine]-3′-carboxamide*** (**7l**)**:** yellow powder, m. p. 196–198°C, yield 85%. IR (KBr) (ν_max_/cm^−1^): 3,567, 3,468, 1,738, 1,695, 1,639, 1,578, 1,468, and 1,298 cm^−1^.^1^H NMR (500 MHz, CDCl_3_): *δ* 1.05 (3 H, s, Me), 1.10 (3 H, s, Me), 2.28 (3 H, s, Me), 2.39 (3 H, s, NMe), 2.82 (1 H, d, ^3^ *J* = 8.0 Hz, CH), 3.04 (1 H, d, ^3^ *J* = 8.0 Hz, CH), 3.67 (1 H, s, CH), 5.68 (1 H, s, CH), 7.06–7.19 (3 H, m, 3 CH), 7.31–7.37 (3 H, m, 3 CH), 7.49 (2 H, d, ^3^ *J*_HH_ = 7.6 Hz, 2 CH), 10.17 (1 H, s, NH), and 10.54 (2 H, s, NH_2_) ppm. ^13^C NMR (125.7 MHz, CDCl_3_): *δ* 21.28, 25.57, 38.06, 40.99, 63.04, 66.79, 70.20, 79.37, 95.19, 114.19, 115.56, 120.92, 123.59, 124.48, 128.84, 129.03, 134.48, 134.65, 135.56, 138.48, 154.50, 160.91, 160.96, and 171.63 ppm. MS, *m/z* (%): 453 (M^+^, 10), 395 (68), and 58 (100). Anal. Calcd for C_27_H_27_N_5_O_2_ (453.55): C, 71.50; H, 6.00; N, 15.44; Found: C, 71.62; H, 6.18; N, 15.63; %.

### Common process for the generation of spiropyrrolopyridine 17a–d

Spiropyridoindolepyrrolidine **7** (2 mmol) prepared in previous reactions was mixed in water with ethylbromopyruvate **15** (2 mmol) and Ag/Fe_3_O_4_/CdO@MWCNT (0.02 g) for 45 min at room temperature. After this, the dimethyl acetylenedicarboxylate **16** (2 mmol) was added to the previous mixture, and the new mixture was stirred for 3 h. The completion of reaction was monitored by TLC, and the solid residue was separated by filtration and cleaned with EtOH and Et_2_O to afford purified spiropyridine **17**.

***4-Ethyl 2,3-dimethyl 1′,4′,4′-trimethyl-3'-(methylcarbamoyl)-7-oxo-5,7-dihydrospiro[pyrido [2′′,3'':4′,5']pyrrolo[3′,2':3,4]pyrido[1,2-a]indole-13,2′-pyrrolidine]-2,3,4-tricarboxylate*** (1**7a**)**:** yellow powder, m. p. 182–184°C, yield 97%. IR (KBr) (ν_max_/cm^−1^): 3,443, 1738, 1,735, 1,697, 1,656, 1,578, 1,487, and 1,298 cm^−1^. ^1^H NMR (500 MHz, CDCl_3_): δ 1.05 (3 H, s, Me), 1.10 (3 H, s, Me), 1.25 (3 H, t, ^3^ *J* = 7.4 Hz, Me), 2.36 (3 H, s, NMe), 2.76 (3 H, s, NMe), 2.83 (1 H, d, ^2^ *J* = 6.2 Hz, CH), 3.07 (1 H, d, ^2^ *J* = 6.2 Hz, CH), 3.65 (1 H, s, CH), 3.84 (3 H, s, MeO), 3.91 (3 H, s, MeO), 4.34 (2 H, q, ^3^ *J* = 7.4 Hz, CH_2_O), 6.29 (1 H, s, CH), 7.08 (1 H, t, ^3^ *J* = 7.3 Hz, CH), 7.23 (1 H, d, ^3^ *J* = 8.6 Hz, CH), 7.38 (1 H, t, ^3^ *J* = 7.6 Hz, CH), 7.46 (1 H, d, ^3^ *J* = 7.6 Hz, CH), 10.24 (1 H, s, NH), and 10.86 (1 H, s, NH) ppm. ^13^C NMR (125.7 MHz, CDCl_3_): *δ* 14.25, 25.55, 25.95, 38.20, 40.51, 52.32, 52.49, 60.81, 63.18, 65.19, 76.03, 101.03, 115.66, 122.78, 122.92, 123.12, 124.23, 126.82, 128.54, 133.61, 133.63, 137.22, 137.23, 141.65, 144.27, 149.74, 162.30, 164.42, 165.83, 166.63, and 172.11 ppm. MS, *m/z* (%): 617 (M^+^, 10), 147 (68), and 31 (100). Anal. Calcd for C_32_H_33_N_5_O_8_ (615.64): C, 62.43; H, 5.40; N, 11.38; Found: C, 62.52; H, 5.53; N, 11.46%.

### Valuation of the antioxidant property via DPPH

As mentioned previously in this research, the antioxidant property of some synthesized spiropyridoindolepyrrolidines such as **7a-7d** was investigated using the DPPH free radical utilizing Shimada et al.’s (1992) procedures. According to the Shimada method, the concentration of spiropyridoindolepyrrolidines **7a-7d** were selected 200–1,000 ppm, in which methanolic solution of DPPH (1 mmol/L) in equivalent volume was added to spiropyridoindolepyrrolidines solution. The new mixture was mixed at room temperature and after 30 min placed in a dark room that absorbance of mixture reached to 517 nm. For comparing the antioxidant activity of synthesized spiropyridoindolepyrrolidine **7a-7d,** butylated hydroxytoluene (BHT) and 2-tertbutylhydroquinone (TBHQ) were utilized and instead of synthesized compounds, methanol (3 ml) was used. For measuring the percentage of inhibition of the DPPH radical trapping experiment, the Yen and Duh (1994) equation was used.

### FRAP process promoted evaluation of the spiropyridoindolpyrrolidine antioxidant activity

Another way for the consideration of spiropyridoindolepyrrolidine antioxidant property is by using the FRAP process that measures the amounts of iron (III) reducing by synthesized spiropyridoindolepyrrolidines **7a-7d,** employing the Yildirim et al. procedure (Yildirim et al., 2001). In this experiment, the spiropyridoindolepyrrolidine solution (1 ml), potassium ferricyanide (2.6 ml), and phosphate buffer (2.6 ml) was used for the evaluation of antioxidant activity according to Yildirim et al.’s procedure. The temperature of the mixture was kept at 55^o^C for 35 min and then trichloroacetic acid (2.5 ml) was added and to the new mixture was stirred for 10 min. Finally, the absorbance of FeCl_3_ (0.6 ml) and supernatant (2.5 ml) mixture in aqueous media (2.6 ml) as a sample was measured at 700 nm. As a result, compounds with a high reducing ability have high amount of absorbance. All computations were carried out three times for the confirmation of calculating. Running the SPSS software version 18.0 is a way to study of variance (ANOVA) for synthesized spiropyridoindolepyrrolidine data analyzing the approved samples and standard variation. Duncan multiple-range experiments were employed for separation with the important extent of 95% (*p* < 0.05).

### Study of the antibacterial activity of the prepared spiropyridoindolepyrrolidine

For this investigation, Persian-type culture collection (PTCC) of Gram-positive and Gram-negative bacteria was prepared in Tehran, Iran, and for this reason, the disk diffusion procedure was utilized. For performing the evaluation of spiropyridoindolepyrrolidine antimicrobial ability, two types of bacterial concentration were similar to McFarland Standard No. 0.5 and were cultured for 16–24 h at 37 C. Two standard drugs such as streptomycin and gentamicin that killed bacteria were utilized. The suspension of bacteria was prepared with a sterile swab cultured on Mueller Hinton agar consistent with McFarland standard No. 0.5 (1.5 × 108 CFU/ml). Then, for the consideration of antibacterial property, spiropyridoindolepyrrolidine (25 μg/ml) was added on sterile blank disks, and the ready sample was placed for 24 h at 37 C in an incubator. The diameter of the inhibition zone was measured and compared to the standard sample.

### The Ag/Fe_3_O_4_/CdO@MWCNT MNC application in the reduction of 4-NP

For this purpose, the mixture of Ag/Fe_3_O_4_/CdO@MWCNT MNCs (0.005 g) and 4-nitrophenol solution (25 ml, 2.5 mM) was stirred for 2 min at room temperature in the beaker, and the newly produced NaBH_4_ (25 ml, 0.25 M) was added to the previous mixture as a reducing agent, which could remove the pollutant in the presence of a catalyst. After adding the aqueous NaBH_4_ to the first mixture, the solution color varied from pale yellow to lemon color. The stirring of the mixture was continued till the mixture became colorless. When the reaction mixture became colorless, for measuring the UV–Vis absorption, 1 ml of the solution was diluted to 25 ml at certain times. The concentration of 4-nitrophenol varied between 200 and 700 nm at room temperature, and it was checked by the UV–Vis absorption spectra. The main point in the catalyst is reusability of it in the same reactions. For the confirmation of this point, the catalyst was removed from the reaction mixture and washed with ethanol and finally dried for reusing in the same reaction.

## 6 Theoretical simulations

### Computational details

Theoretical studies were carried out by the Gaussian 09 package (Frisch et al., 2009). The electronic structure and geometries, Mulliken atomic charges, and vibrational frequencies of 6a-6d compounds were computed within the density functional theory DFT/B_3_LYP method with 6–311++G** basis set. The global reactivity descriptors such as ionization energy (I), electron affinity (A), chemical potential (μ), chemical hardness (η), molecular electrophilicity (w), and chemical softness were computed from the energies of HOMO and LUMO using equations (1)-(6) (Umadevi and Lalitha, 2012), and the values reactivity descriptors were obtained using Multiwfn 3.7 code Lu et al., 2012). For a good agreement between theoretical and experimental data, the calculated frequencies were scaled using the Pulay scaled quantum mechanical force field methodology.

$$\text{I}=-{\text{E}}_{HOMO},$$ (1)

$$\text{A}=-{\text{E}}_{LUMO},$$ (2)

$$\text{μ}=\left({\text{E}}_{LUMO}+{\text{E}}_{HOMO}\right)/2,$$ (3)

$$\text{η}=\left({\text{E}}_{LUMO}-{\text{E}}_{HOMO}\right)/2,$$ (4)

S=1/η, (5)

$$\text{ω}=\mu^{2}/2\eta .$$ (6)

## Data Availability

The original contributions presented in the study are included in the article/Supplementary Material. Further inquiries can be directed to the corresponding author.
