# Supplementary material for: Six-component synthesis and biological activity of novel spiropyridoindolepyrrolidine derivatives: A combined experimental and theoretical investigation
Source: Front Chem. 2022 Sep 21;10:949205. doi: 10.3389/fchem.2022.949205 (PMC9559721; doi:10.3389/fchem.2022.949205)

Supplementary Material

Six Component Synthesis and Biological activity of Novel Spiropyridoindolepyrrolidine Derivatives: A Combined Experimental and Theoretical Investigation

Zinatossadat Hossaini[1], Marziyeh Mohammadi[2] and Fatemeh Sheikholeslami-Farahani[3]

[1]Department of Chemistry, Qaemshahr Branch, Islamic Azad University, Qaemshahr, Iran

[2]Department of Chemistry, Faculty of Science, Vali-e-Asr University of Rafsanjan, Rafsanjan 77176, Iran.Email: m.mohammadi@vru.ac.ir

[3]Department of Chemistry, Firoozkooh Branch, Islamic Azad University, Firoozkooh, Iran

***Corresponding author:** E-mail addresses: zshossaini@yahoo.com

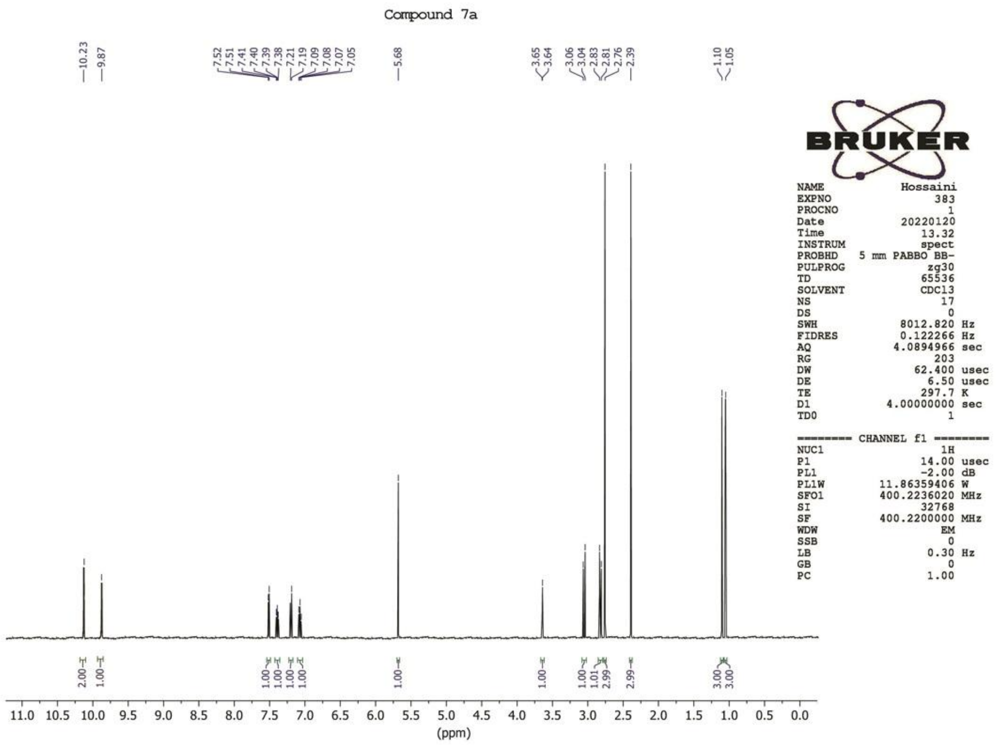
Compound 7a
10.23
9.87
7.52
7.51
7.41
7.40
7.39
7.38
7.21
7.19
7.09
7.08
7.07
7.05
5.68
3.65
3.64
3.06
3.04
2.83
2.81
2.76
2.39
1.10
1.05
BRUKER
NAME Hossaini
EXPNO 383
PROCNO 1
Date 20220120
Time 13.32
INSTRUM spect
PROBHD 5 mm PABBO BB-
PULPROG zg30
TD 65536
SOLVENT CDCl3
NS 17
DS 0
SWH 8012.820 Hz
FIDRES 0.122266 Hz
AQ 4.0894966 sec
RG 203
DW 62.400 usec
DE 6.50 usec
TE 297.7 K
D1 4.00000000 sec
TD0 1
======== CHANNEL f1 ========
NUC1 1H
P1 14.00 usec
PL1 -2.00 dB
PL1W 11.86359406 W
SFO1 400.2236020 MHz
SI 32768
SF 400.2200000 MHz
WDW EM
SSB 0
LB 0.30 Hz
GB 0
PC 1.00
2.00
1.00
1.00
1.00
1.00
1.00
1.00
1.00
1.00
1.01
2.99
2.99
3.00
3.00
11.0 10.5 10.0 9.5 9.0 8.5 8.0 7.5 7.0 6.5 6.0 5.5 5.0 4.5 4.0 3.5 3.0 2.5 2.0 1.5 1.0 0.5 0.0
(ppm)

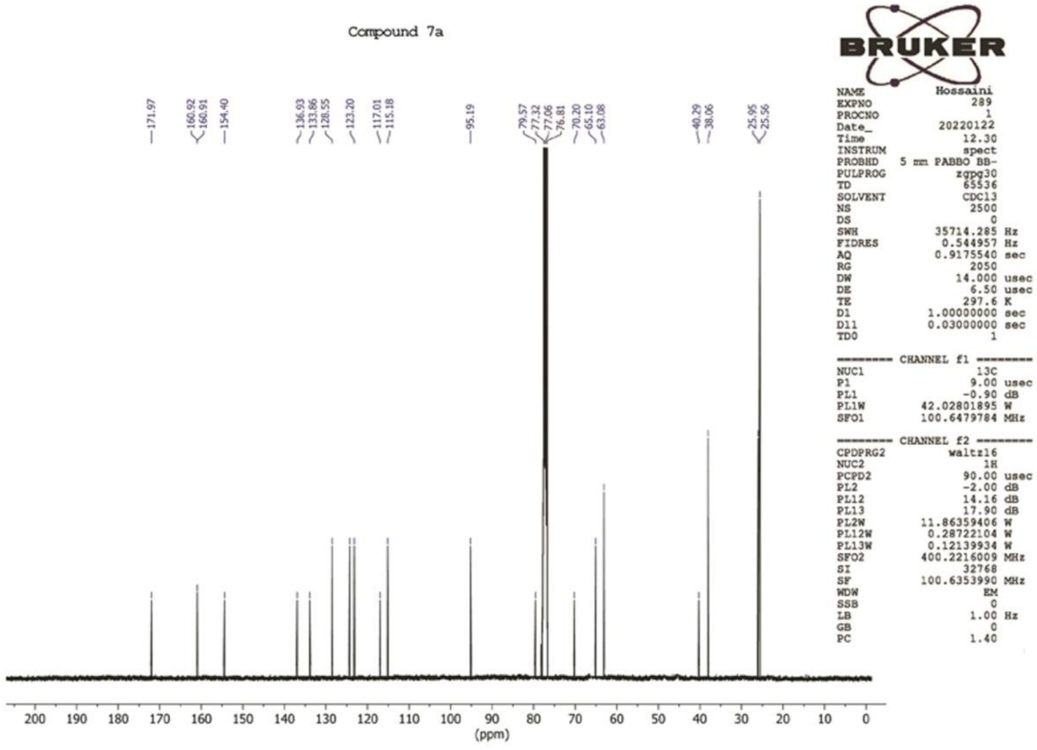
Compound 7a
171.97
160.92
160.91
154.40
136.93
133.86
128.55
123.20
117.01
115.18
95.19
79.57
77.32
77.06
76.81
70.20
65.10
63.08
40.29
38.06
25.95
25.56
BRUKER
NAME Hossaini
EXPNO 289
PROCNO 1
Date_ 20220122
Time 12.30
INSTRUM spect
PROBHD 5 mm PABBO BB-
PULPROG zgpg30
TD 65536
SOLVENT CDCl3
NS 2500
DS 0
SWH 35714.285 Hz
FIDRES 0.544957 Hz
AQ 0.9175540 sec
RG 2050
DW 14.000 usec
DE 6.50 usec
TE 297.6 K
D1 1.00000000 sec
D11 0.03000000 sec
TD0 1
======== CHANNEL f1 ========
NUC1 13C
P1 9.00 usec
PL1 -0.90 dB
PL1W 42.02801895 W
SFO1 100.6479784 MHz
======== CHANNEL f2 ========
CPDPRG2 waltz16
NUC2 1H
PCPD2 90.00 usec
PL2 -2.00 dB
PL12 14.16 dB
PL13 17.90 dB
PL2W 11.86359406 W
PL12W 0.28722104 W
PL13W 0.12139934 W
SFO2 400.2216009 MHz
SI 32768
SF 100.6353990 MHz
WDW EM
SSB 0
LB 1.00 Hz
GB 0
PC 1.40
200 190 180 170 160 150 140 130 120 110 100 90 80 70 60 50 40 30 20 10 0
(ppm)

Compound 7b

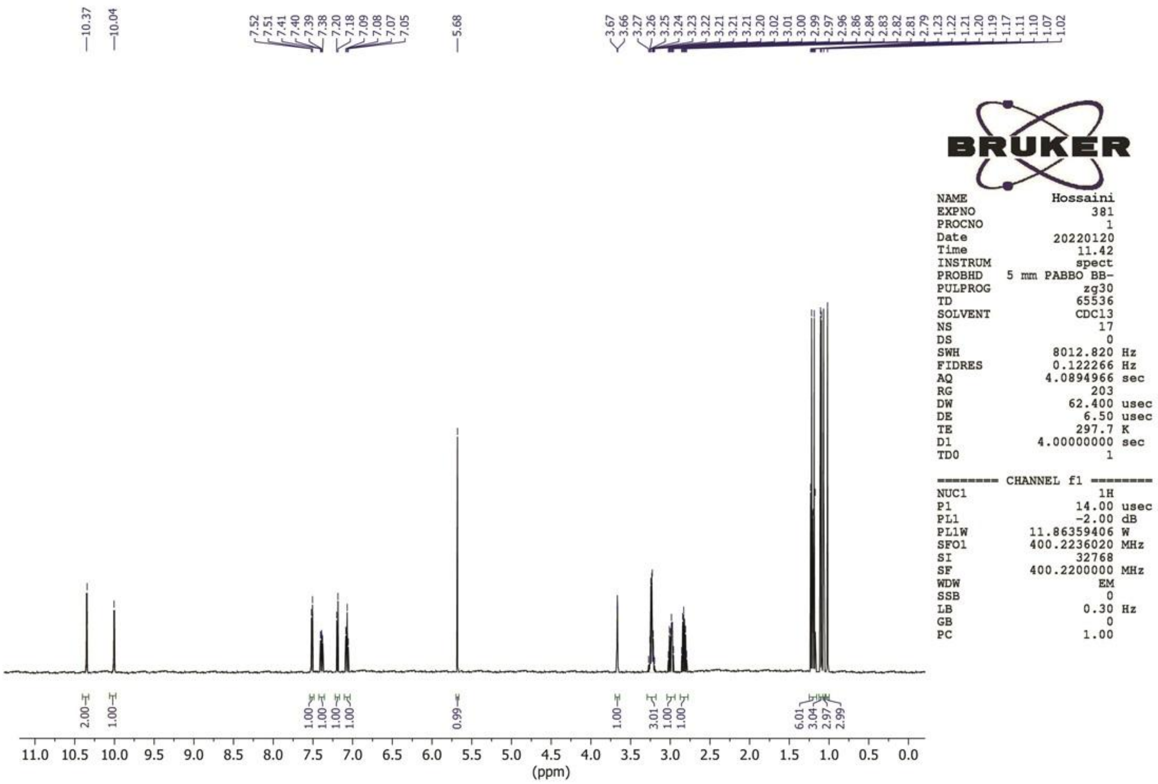

Compound 7b

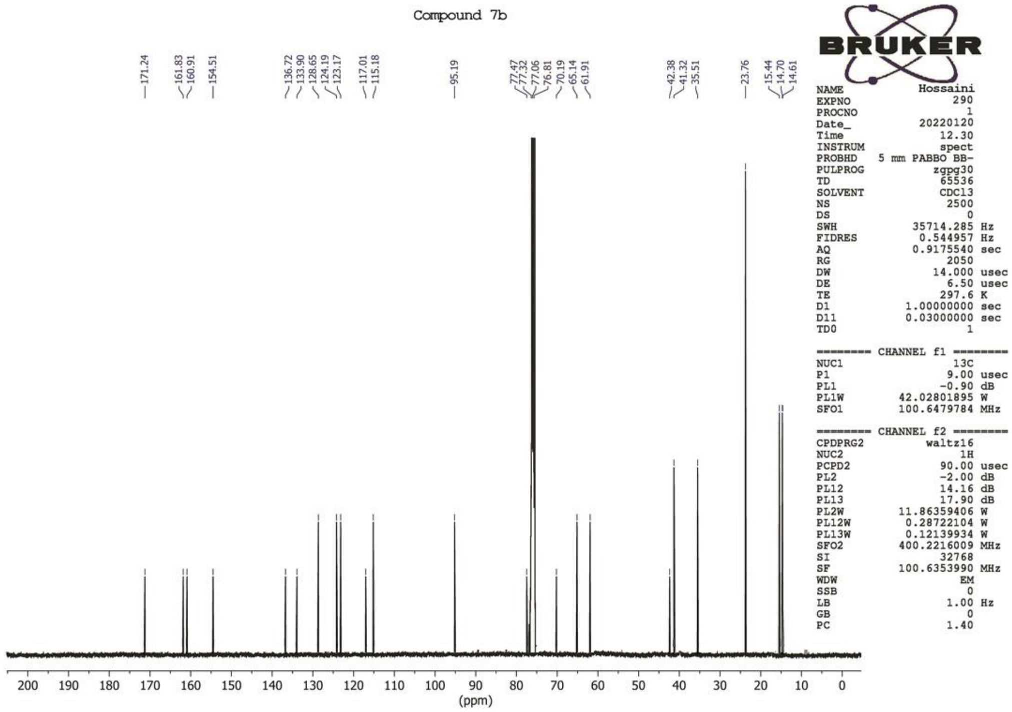

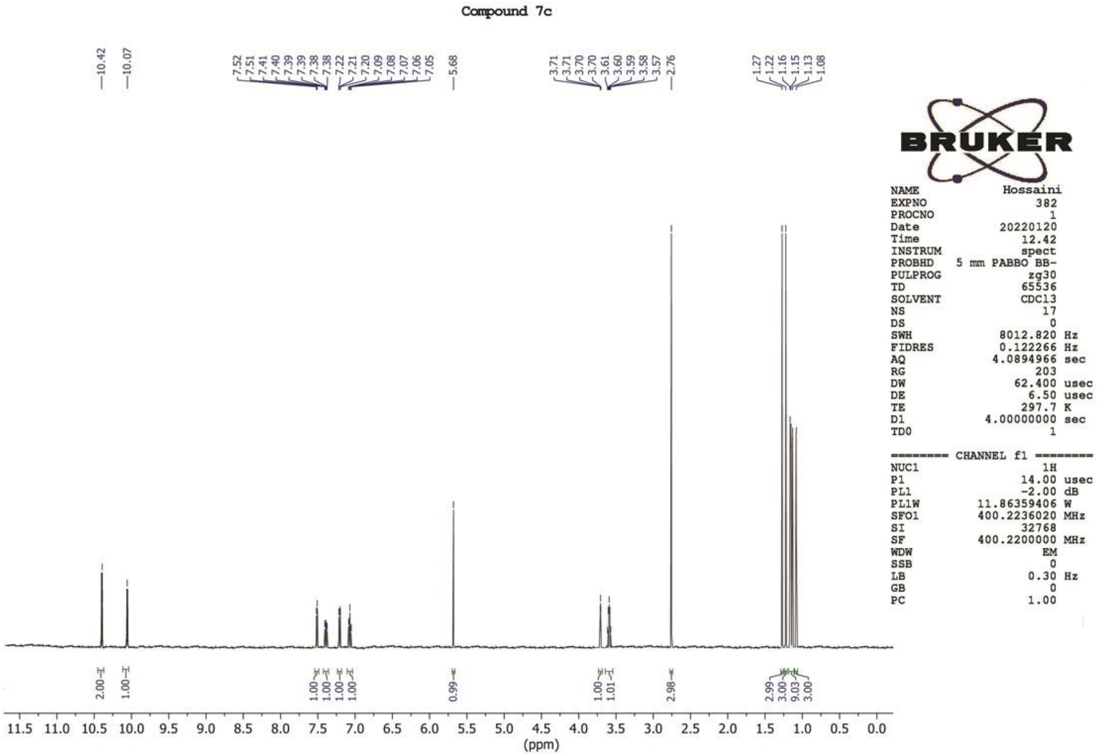
Compound 7c
10.42
10.07
7.52
7.51
7.41
7.40
7.39
7.39
7.38
7.38
7.22
7.21
7.20
7.09
7.08
7.07
7.06
7.05
5.68
3.71
3.71
3.70
3.70
3.61
3.60
3.59
3.58
3.57
2.76
1.27
1.22
1.16
1.15
1.13
1.08
BRUKER
NAME Hossaini
EXPNO 382
PROCNO 1
Date 20220120
Time 12.42
INSTRUM spect
PROBHD 5 mm PABBO BB-
PULPROG zg30
TD 65536
SOLVENT CDCl3
NS 17
DS 0
SWH 8012.820 Hz
FIDRES 0.122266 Hz
AQ 4.0894966 sec
RG 203
DW 62.400 usec
DE 6.50 usec
TE 297.7 K
D1 4.00000000 sec
TD0 1
======== CHANNEL f1 ========
NUC1 1H
P1 14.00 usec
PL1 -2.00 dB
PL1W 11.86359406 W
SFO1 400.2236020 MHz
SI 32768
SF 400.2200000 MHz
WDW EM
SSB 0
LB 0.30 Hz
GB 0
PC 1.00
2.00
1.00
1.00
1.00
1.00
1.00
0.99
1.00
1.01
2.98
2.99
3.00
9.03
3.00
11.5 11.0 10.5 10.0 9.5 9.0 8.5 8.0 7.5 7.0 6.5 6.0 5.5 5.0 4.5 4.0 3.5 3.0 2.5 2.0 1.5 1.0 0.5 0.0
(ppm)

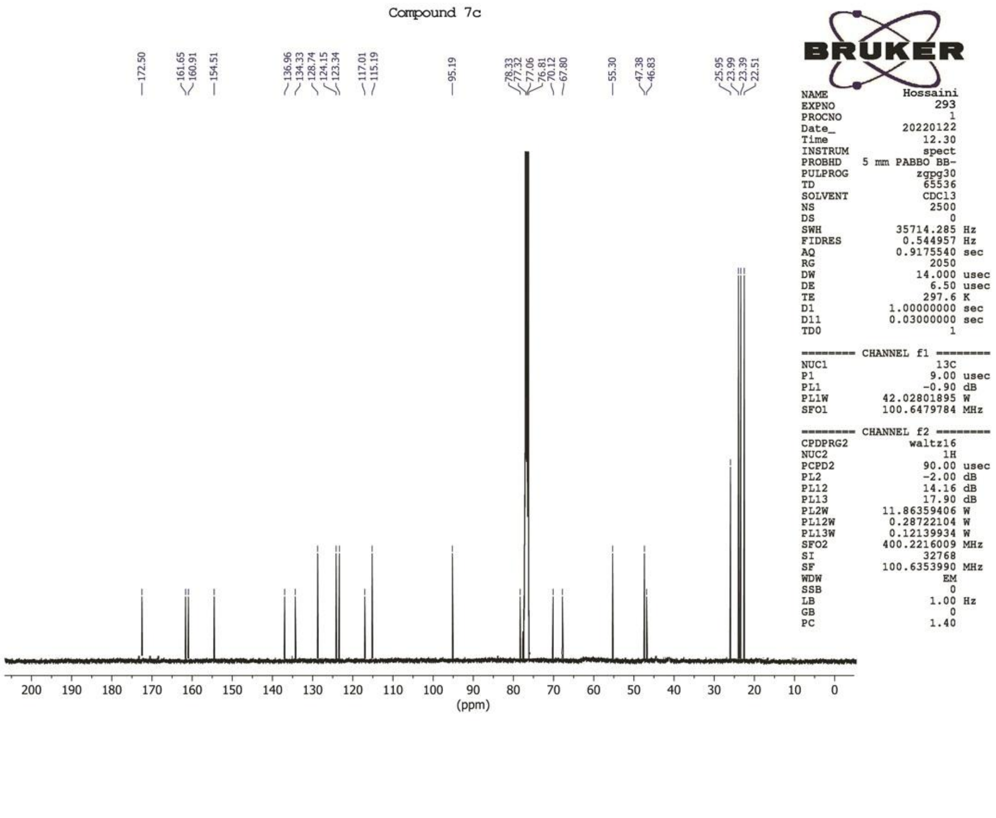
Compound 7c
172.50
161.65
160.91
154.51
136.96
134.33
128.74
124.15
123.34
117.01
115.19
95.19
78.33
77.32
77.06
76.81
70.12
67.80
55.30
47.38
46.83
25.95
23.99
23.39
22.51
BRUKER
NAME Hossaini
EXPNO 293
PROCNO 1
Date_ 20220122
Time 12.30
INSTRUM spect
PROBHD 5 mm PABBO BB-
PULPROG zgpg30
TD 65536
SOLVENT CDCl3
NS 2500
DS 0
SWH 35714.285 Hz
FIDRES 0.544957 Hz
AQ 0.9175540 sec
RG 2050
DW 14.000 usec
DE 6.50 usec
TE 297.6 K
D1 1.00000000 sec
D11 0.03000000 sec
TD0 1
======== CHANNEL f1 ========
NUC1 13C
P1 9.00 usec
PL1 -0.90 dB
PL1W 42.02801895 W
SFO1 100.6479784 MHz
======== CHANNEL f2 ========
CPDPRG2 waltz16
NUC2 1H
PCPD2 90.00 usec
PL2 -2.00 dB
PL12 14.16 dB
PL13 17.90 dB
PL2W 11.86359406 W
PL12W 0.28722104 W
PL13W 0.12139934 W
SFO2 400.2216009 MHz
SI 32768
SF 100.6353990 MHz
WDW EM
SSB 0
LB 1.00 Hz
GB 0
PC 1.40
200 190 180 170 160 150 140 130 120 110 100 90 80 70 60 50 40 30 20 10 0
(ppm)

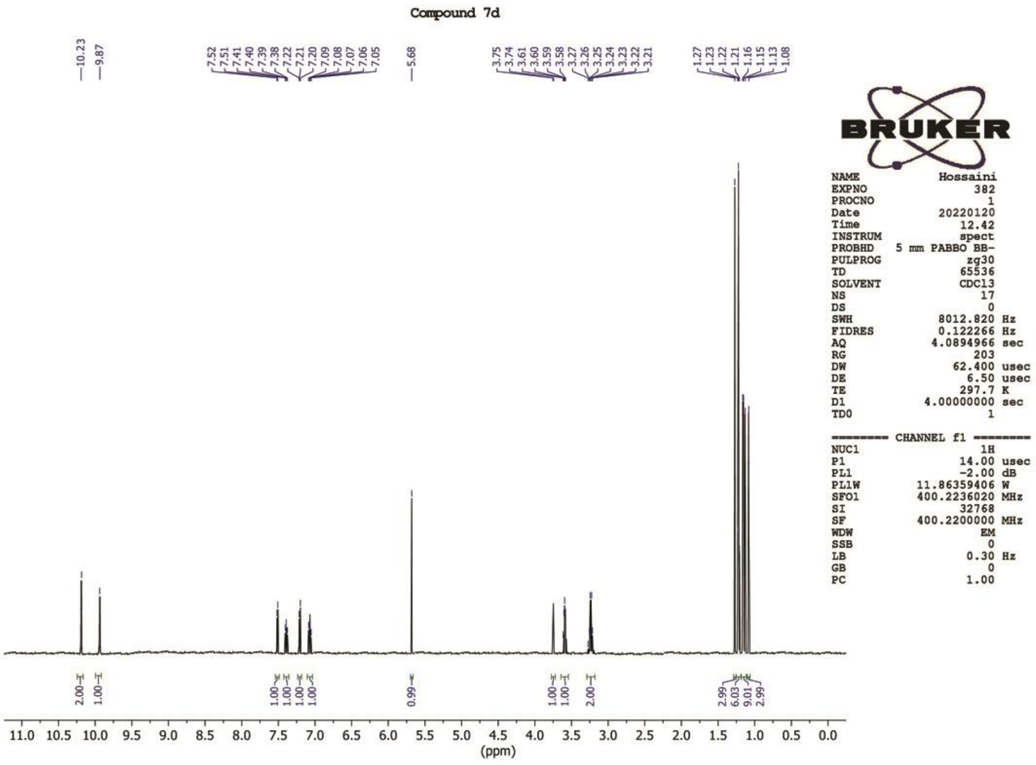
Compound 7d
NAME Hossaini
EXPNO 382
PROCNO 1
Date 20220120
Time 12.42
INSTRUM spect
PROBHD 5 mm PABBO BB-
PULPROG zg30
TD 65536
SOLVENT CDCl3
NS 17
DS 0
SWH 8012.820 Hz
FIDRES 0.122266 Hz
AQ 4.0894966 sec
RG 203
DW 62.400 usec
DE 6.50 usec
TE 297.7 K
D1 4.00000000 sec
TD0 1
======== CHANNEL f1 ========
NUC1 1H
P1 14.00 usec
PL1 -2.00 dB
PL1W 11.86359406 W
SFO1 400.2236020 MHz
SI 32768
SF 400.2200000 MHz
WDW EM
SSB 0
LB 0.30 Hz
GB 0
PC 1.00
(ppm)

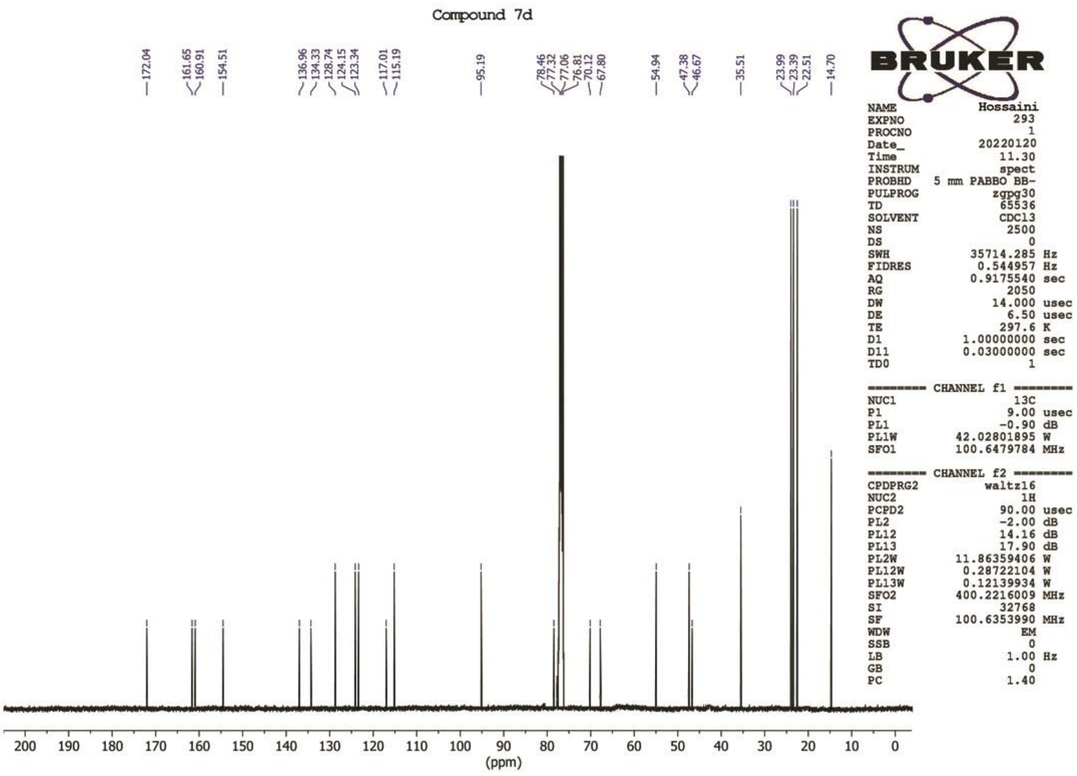
Compound 7d
NAME Hossaini
EXPNO 293
PROCNO 1
Date_ 20220120
Time 11.30
INSTRUM spect
PROBHD 5 mm PABBO BB-
PULPROG zgpg30
TD 65536
SOLVENT CDCl3
NS 2500
DS 0
SWH 35714.285 Hz
FIDRES 0.544957 Hz
AQ 0.9175540 sec
RG 2050
DW 14.000 usec
DE 6.50 usec
TE 297.6 K
D1 1.00000000 sec
D11 0.03000000 sec
TD0 1
======== CHANNEL f1 ========
NUC1 13C
P1 9.00 usec
PL1 -0.90 dB
PL1W 42.02801895 W
SFO1 100.6479784 MHz
======== CHANNEL f2 ========
CPDPRG2 waltz16
NUC2 1H
PCPD2 90.00 usec
PL2 -2.00 dB
PL12 14.16 dB
PL13 17.90 dB
PL2W 11.86359406 W
PL12W 0.28722104 W
PL13W 0.12139934 W
SFO2 400.2216009 MHz
SI 32768
SF 100.6353990 MHz
WDW EM
SSB 0
LB 1.00 Hz
GB 0
PC 1.40
(ppm)

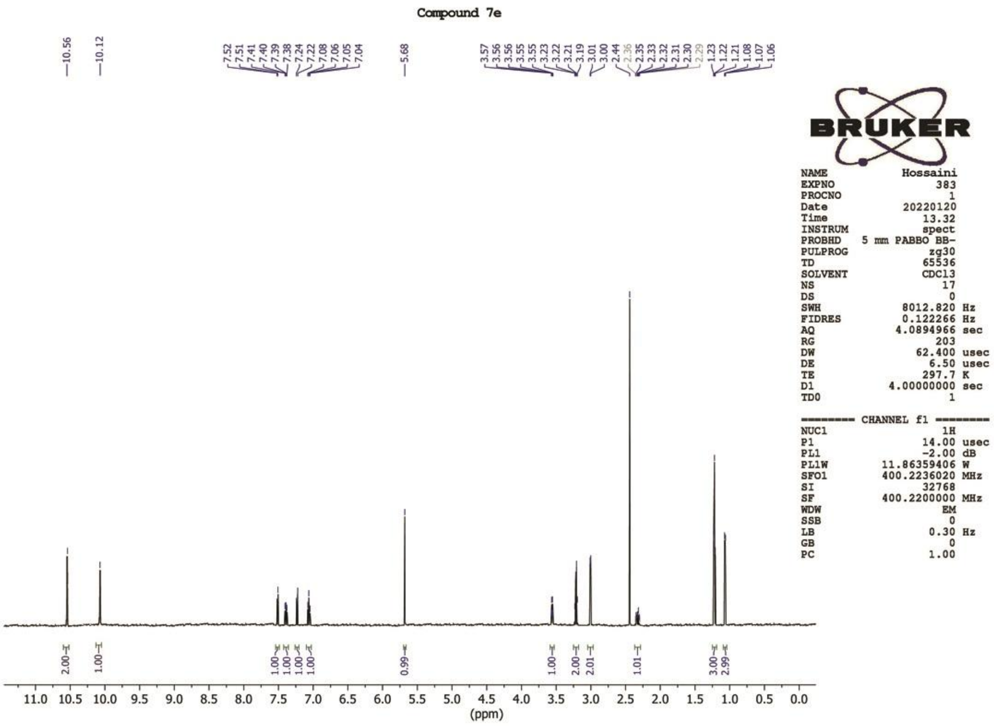
Compound 7e
BRUKER
NAME Hossaini
EXPNO 383
PROCNO 1
Date 20220120
Time 13.32
INSTRUM spect
PROBHD 5 mm PABBO BB-
PULPROG zg30
TD 65536
SOLVENT CDCl3
NS 17
DS 0
SWH 8012.820 Hz
FIDRES 0.122266 Hz
AQ 4.0894966 sec
RG 203
DW 62.400 usec
DE 6.50 usec
TE 297.7 K
D1 4.00000000 sec
TD0 1
======== CHANNEL f1 ========
NUC1 1H
P1 14.00 usec
PL1 -2.00 dB
PL1W 11.86359406 W
SFO1 400.2236020 MHz
SI 32768
SF 400.2200000 MHz
WDW EM
SSB 0
LB 0.30 Hz
GB 0
PC 1.00
(ppm)

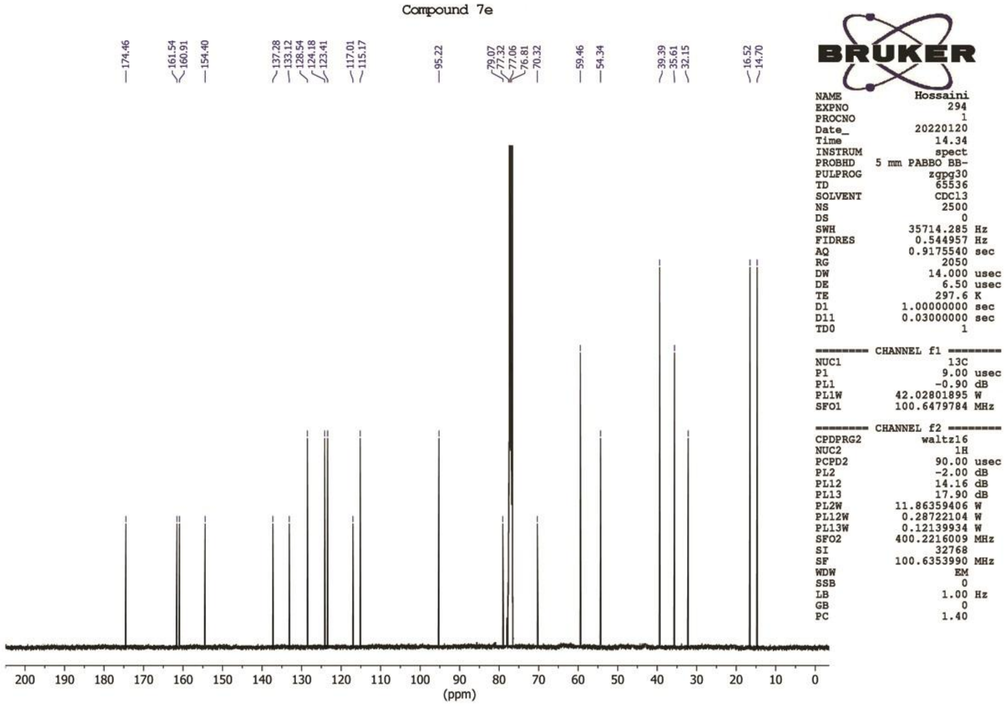
Compound 7e
BRUKER
NAME Hossaini
EXPNO 294
PROCNO 1
Date_ 20220120
Time 14.34
INSTRUM spect
PROBHD 5 mm PABBO BB-
PULPROG zgpg30
TD 65536
SOLVENT CDCl3
NS 2500
DS 0
SWH 35714.285 Hz
FIDRES 0.544957 Hz
AQ 0.9175540 sec
RG 2050
DW 14.000 usec
DE 6.50 usec
TE 297.6 K
D1 1.00000000 sec
D11 0.03000000 sec
TD0 1
======== CHANNEL f1 ========
NUC1 13C
P1 9.00 usec
PL1 -0.90 dB
PL1W 42.02801895 W
SFO1 100.6479784 MHz
======== CHANNEL f2 ========
CPDPRG2 waltz16
NUC2 1H
PCPD2 90.00 usec
PL2 -2.00 dB
PL12 14.16 dB
PL13 17.90 dB
PL2W 11.86359406 W
PL12W 0.28722104 W
PL13W 0.12139934 W
SFO2 400.2216009 MHz
SI 32768
SF 100.6353990 MHz
WDW EM
SSB 0
LB 1.00 Hz
GB 0
PC 1.40
(ppm)

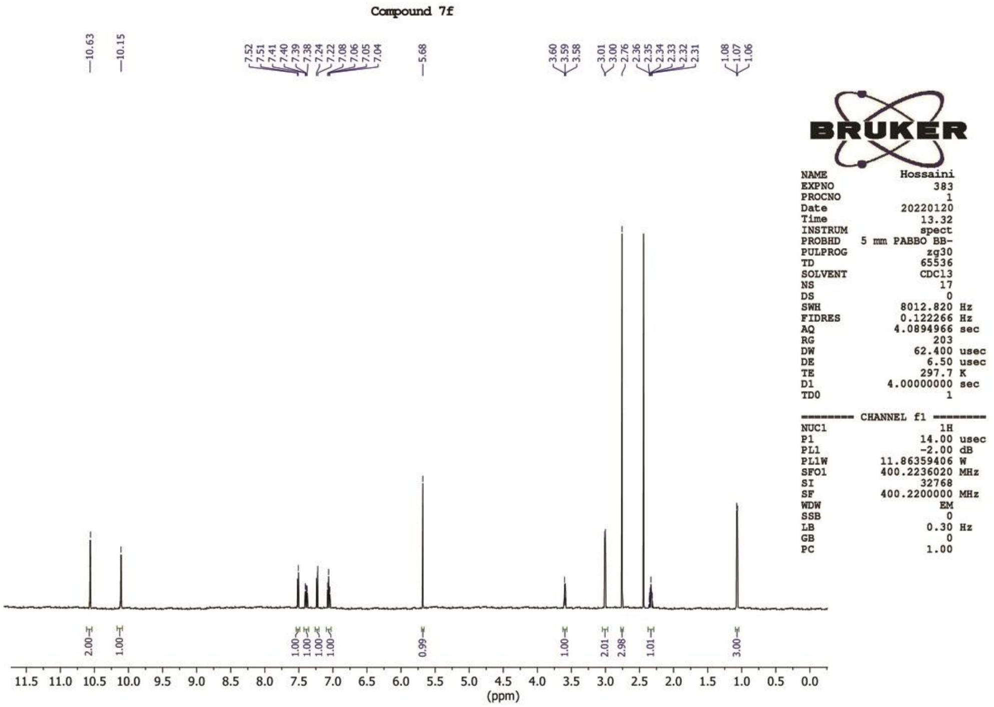
Compound 7f
BRUKER
NAME Hossaini
EXPNO 383
PROCNO 1
Date 20220120
Time 13.32
INSTRUM spect
PROBHD 5 mm PABBO BB-
PULPROG zg30
TD 65536
SOLVENT CDCl3
NS 17
DS 0
SWH 8012.820 Hz
FIDRES 0.122266 Hz
AQ 4.0894966 sec
RG 203
DW 62.400 usec
DE 6.50 usec
TE 297.7 K
D1 4.00000000 sec
TD0 1
======== CHANNEL f1 ========
NUC1 1H
P1 14.00 usec
PL1 -2.00 dB
PL1W 11.86359406 W
SFO1 400.2236020 MHz
SI 32768
SF 400.2200000 MHz
WDW EM
SSB 0
LB 0.30 Hz
GB 0
PC 1.00
(ppm)

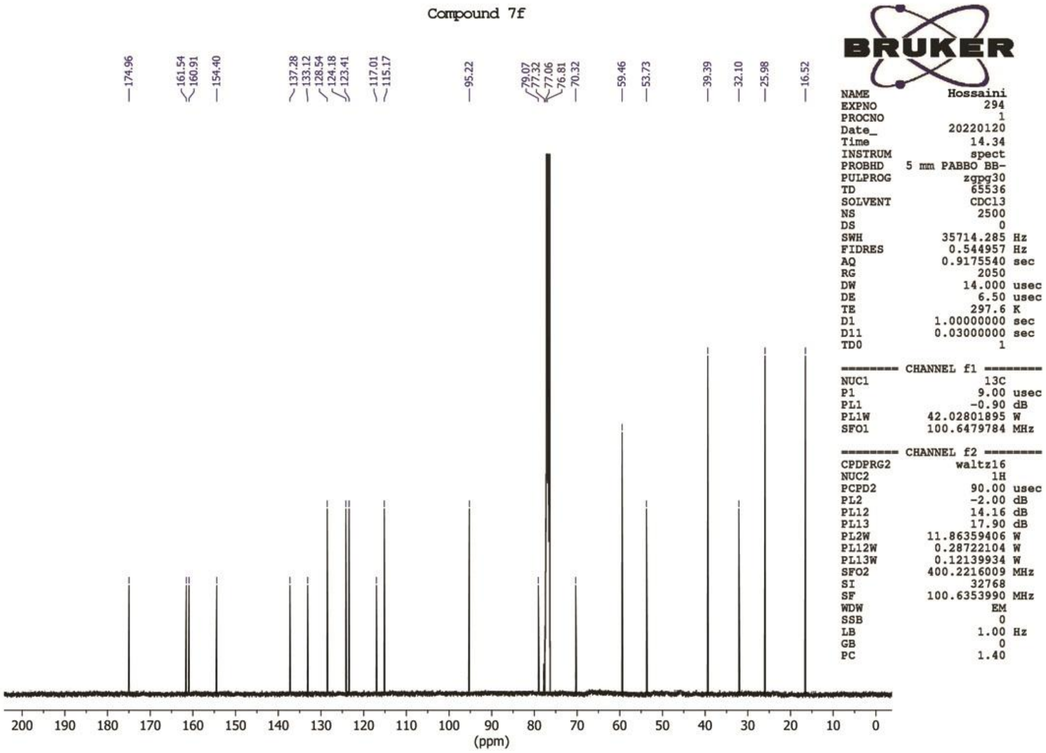
Compound 7f
BRUKER
NAME Hossaini
EXPNO 294
PROCNO 1
Date_ 20220120
Time 14.34
INSTRUM spect
PROBHD 5 mm PABBO BB-
PULPROG zgpg30
TD 65536
SOLVENT CDCl3
NS 2500
DS 0
SWH 35714.285 Hz
FIDRES 0.544957 Hz
AQ 0.9175540 sec
RG 2050
DW 14.000 usec
DE 6.50 usec
TE 297.6 K
D1 1.00000000 sec
D11 0.03000000 sec
TD0 1
======== CHANNEL f1 ========
NUC1 13C
P1 9.00 usec
PL1 -0.90 dB
PL1W 42.02801895 W
SFO1 100.6479784 MHz
======== CHANNEL f2 ========
CPDPRG2 waltz16
NUC2 1H
PCPD2 90.00 usec
PL2 -2.00 dB
PL12 14.16 dB
PL13 17.90 dB
PL2W 11.86359406 W
PL12W 0.28722104 W
PL13W 0.12139934 W
SFO2 400.2216009 MHz
SI 32768
SF 100.6353990 MHz
WDW EM
SSB 0
LB 1.00 Hz
GB 0
PC 1.40
(ppm)

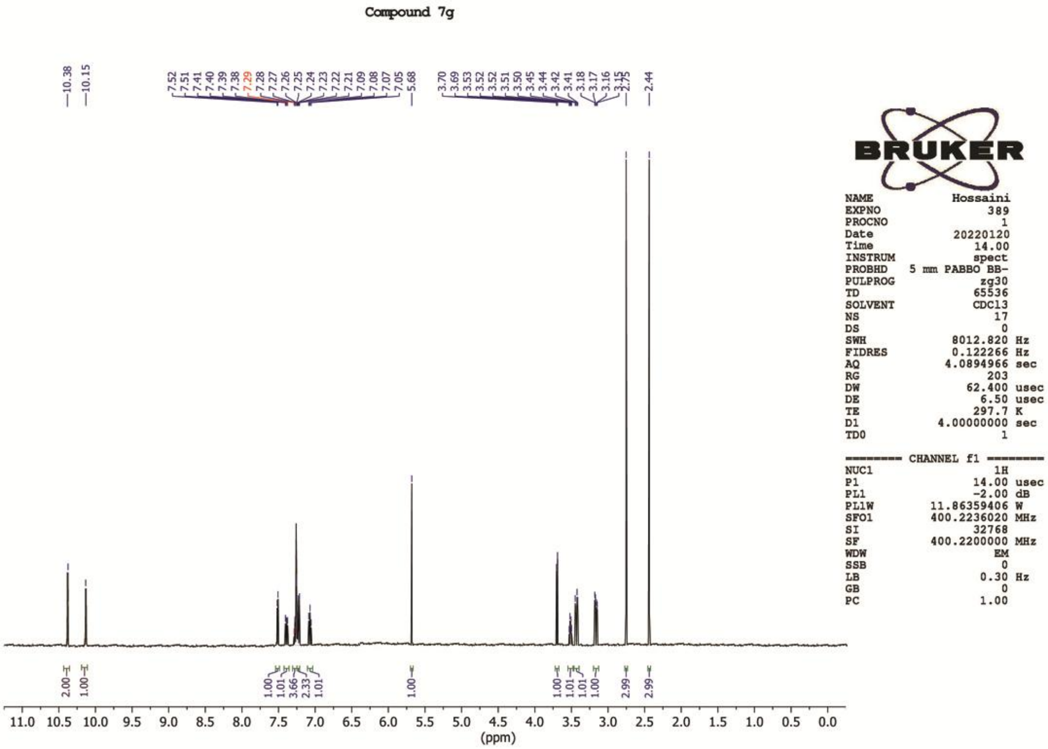
Compound 7g
10.38
10.15
7.52 7.51 7.41 7.40 7.39 7.38 7.29 7.28 7.27 7.26 7.25 7.24 7.23 7.22 7.21 7.09 7.08 7.07 7.05
5.68
3.70 3.69 3.53 3.52 3.52 3.51 3.50 3.45 3.44 3.42 3.41 3.18 3.17 3.16 3.15
2.75
2.44
2.00 1.00 1.00 1.01 3.66 2.33 1.01 1.00 1.00 1.01 1.01 1.00 2.99 2.99
11.0 10.5 10.0 9.5 9.0 8.5 8.0 7.5 7.0 6.5 6.0 5.5 5.0 4.5 4.0 3.5 3.0 2.5 2.0 1.5 1.0 0.5 0.0
(ppm)
BRUKER
NAME Hossaini
EXPNO 389
PROCNO 1
Date 20220120
Time 14.00
INSTRUM spect
PROBHD 5 mm PABBO BB-
PULPROG zg30
TD 65536
SOLVENT CDCl3
NS 17
DS 0
SWH 8012.820 Hz
FIDRES 0.122266 Hz
AQ 4.0894966 sec
RG 203
DW 62.400 usec
DE 6.50 usec
TE 297.7 K
D1 4.00000000 sec
TD0 1
======== CHANNEL f1 ========
NUC1 1H
P1 14.00 usec
PL1 -2.00 dB
PL1W 11.86359406 W
SFO1 400.2236020 MHz
SI 32768
SF 400.2200000 MHz
WDW EM
SSB 0
LB 0.30 Hz
GB 0
PC 1.00

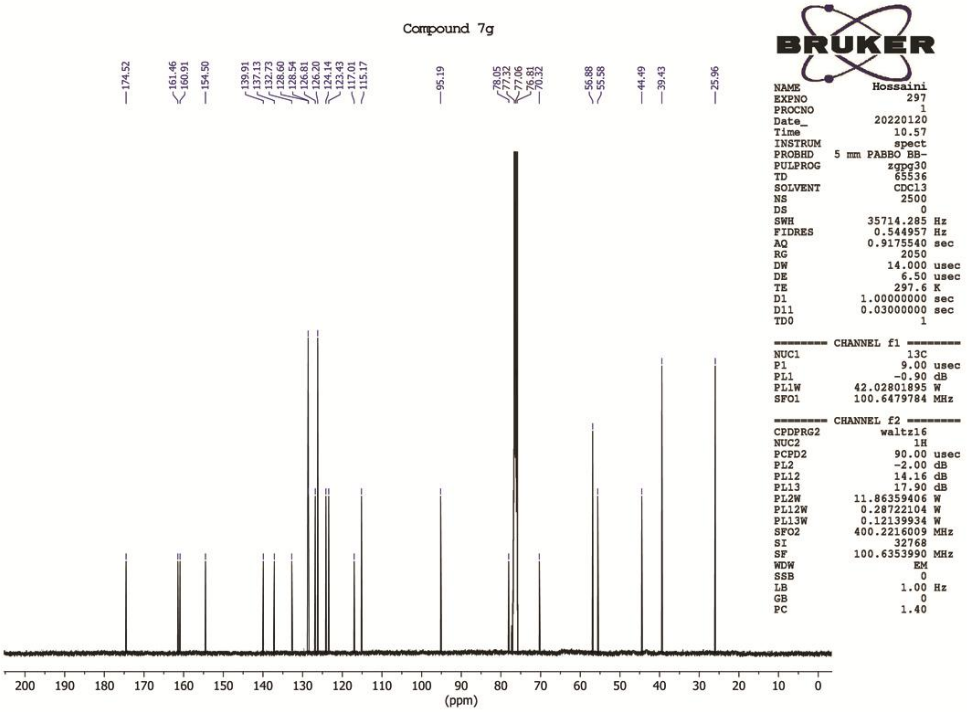
Compound 7g
174.52
161.46 160.91
154.50
139.91 137.13 132.73 128.60 128.54 126.81 126.20 124.14 123.43 117.01 115.17
95.19
78.05 77.32 77.06 76.81 70.32
56.88 55.58
44.49
39.43
25.96
200 190 180 170 160 150 140 130 120 110 100 90 80 70 60 50 40 30 20 10 0
(ppm)
BRUKER
NAME Hossaini
EXPNO 297
PROCNO 1
Date_ 20220120
Time 10.57
INSTRUM spect
PROBHD 5 mm PABBO BB-
PULPROG zgpg30
TD 65536
SOLVENT CDCl3
NS 2500
DS 0
SWH 35714.285 Hz
FIDRES 0.544957 Hz
AQ 0.9175540 sec
RG 2050
DW 14.000 usec
DE 6.50 usec
TE 297.6 K
D1 1.00000000 sec
D11 0.03000000 sec
TD0 1
======== CHANNEL f1 ========
NUC1 13C
P1 9.00 usec
PL1 -0.90 dB
PL1W 42.02801895 W
SFO1 100.6479784 MHz
======== CHANNEL f2 ========
CPDPRG2 waltz16
NUC2 1H
PCPD2 90.00 usec
PL2 -2.00 dB
PL12 14.16 dB
PL13 17.90 dB
PL2W 11.86359406 W
PL12W 0.28722104 W
PL13W 0.12139934 W
SFO2 400.2216009 MHz
SI 32768
SF 100.6353990 MHz
WDW EM
SSB 0
LB 1.00 Hz
GB 0
PC 1.40

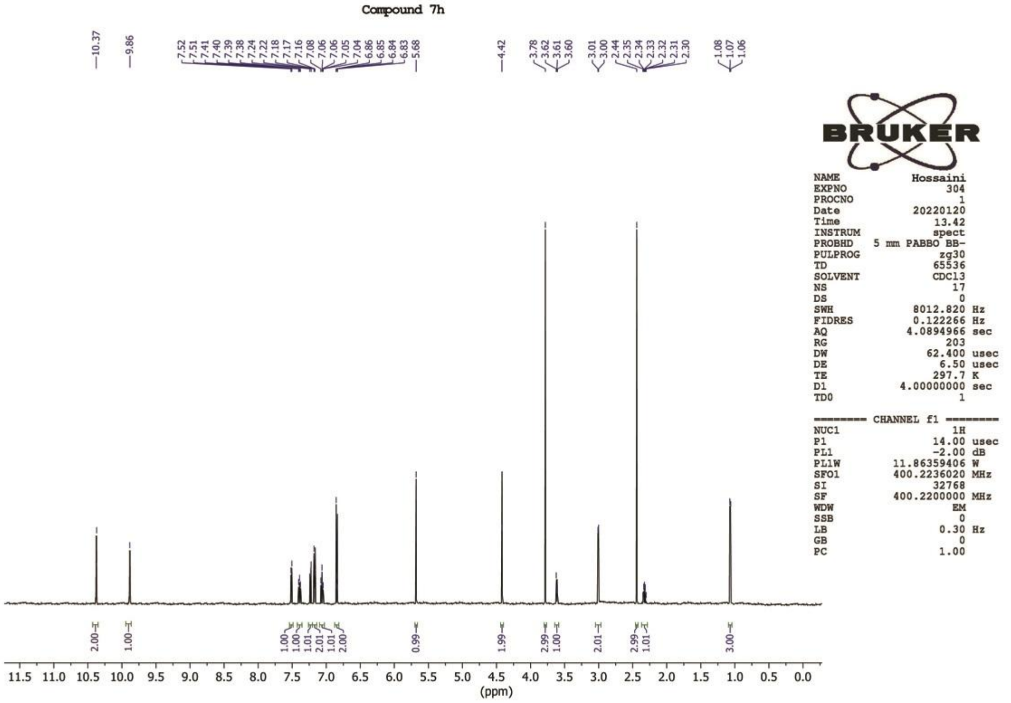
Compound 7h
10.37
9.86
7.52 7.51 7.41 7.40 7.39 7.38 7.24 7.22 7.18 7.17 7.16 7.08 7.06 7.06 7.05 7.04 6.86 6.85 6.84 6.83 5.68
4.42
3.78 3.62 3.61 3.60
3.01 3.00 2.44 2.35 2.34 2.33 2.32 2.31 2.30
1.08 1.07 1.06
BRUKER
NAME Hossaini
EXPNO 304
PROCNO 1
Date 20220120
Time 13.42
INSTRUM spect
PROBHD 5 mm PABBO BB-
PULPROG zg30
TD 65536
SOLVENT CDCl3
NS 17
DS 0
SWH 8012.820 Hz
FIDRES 0.122266 Hz
AQ 4.0894966 sec
RG 203
DW 62.400 usec
DE 6.50 usec
TE 297.7 K
D1 4.00000000 sec
TD0 1
CHANNEL f1
NUC1 1H
P1 14.00 usec
PL1 -2.00 dB
PL1W 11.86359406 W
SFO1 400.2236020 MHz
SI 32768
SF 400.2200000 MHz
WDW EM
SSB 0
LB 0.30 Hz
GB 0
PC 1.00
2.00 1.00 1.00 1.00 2.01 2.01 1.01 2.00 0.99 1.99 2.99 1.00 2.01 2.99 1.01 3.00
11.5 11.0 10.5 10.0 9.5 9.0 8.5 8.0 7.5 7.0 6.5 6.0 5.5 5.0 4.5 4.0 3.5 3.0 2.5 2.0 1.5 1.0 0.5 0.0
(ppm)

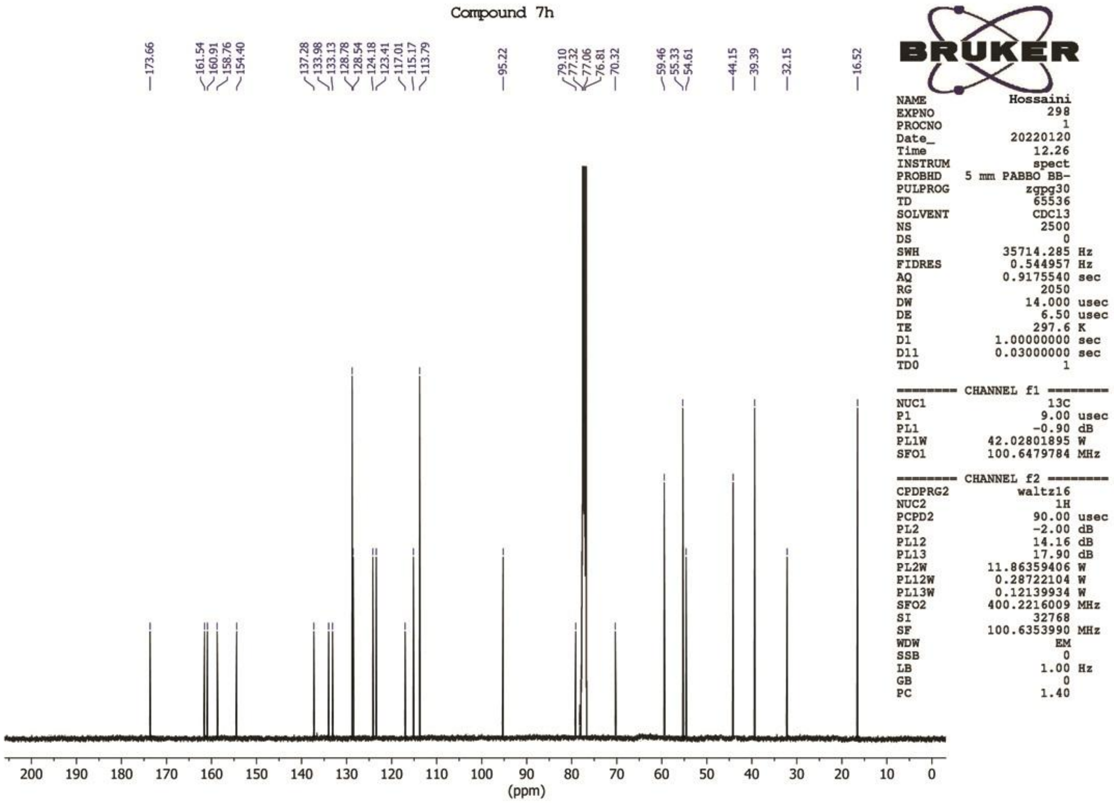
Compound 7h
173.66
161.54 160.91 158.76 154.40
137.28 133.98 133.13 128.78 128.54 124.18 123.41 117.01 115.17 113.79
95.22
79.10 77.32 77.06 76.81 70.32
59.46 55.33 54.61
44.15
39.39
32.15
16.52
BRUKER
NAME Hossaini
EXPNO 298
PROCNO 1
Date_ 20220120
Time 12.26
INSTRUM spect
PROBHD 5 mm PABBO BB-
PULPROG zgpg30
TD 65536
SOLVENT CDCl3
NS 2500
DS 0
SWH 35714.285 Hz
FIDRES 0.544957 Hz
AQ 0.9175540 sec
RG 2050
DW 14.000 usec
DE 6.50 usec
TE 297.6 K
D1 1.00000000 sec
D11 0.03000000 sec
TD0 1
CHANNEL f1
NUC1 13C
P1 9.00 usec
PL1 -0.90 dB
PL1W 42.02801895 W
SFO1 100.6479784 MHz
CHANNEL f2
CPDPRG2 waltz16
NUC2 1H
PCPD2 90.00 usec
PL2 -2.00 dB
PL12 14.16 dB
PL13 17.90 dB
PL2W 11.86359406 W
PL12W 0.28722104 W
PL13W 0.12139934 W
SFO2 400.2216009 MHz
SI 32768
SF 100.6353990 MHz
WDW EM
SSB 0
LB 1.00 Hz
GB 0
PC 1.40
200 190 180 170 160 150 140 130 120 110 100 90 80 70 60 50 40 30 20 10 0
(ppm)

Compound 7i

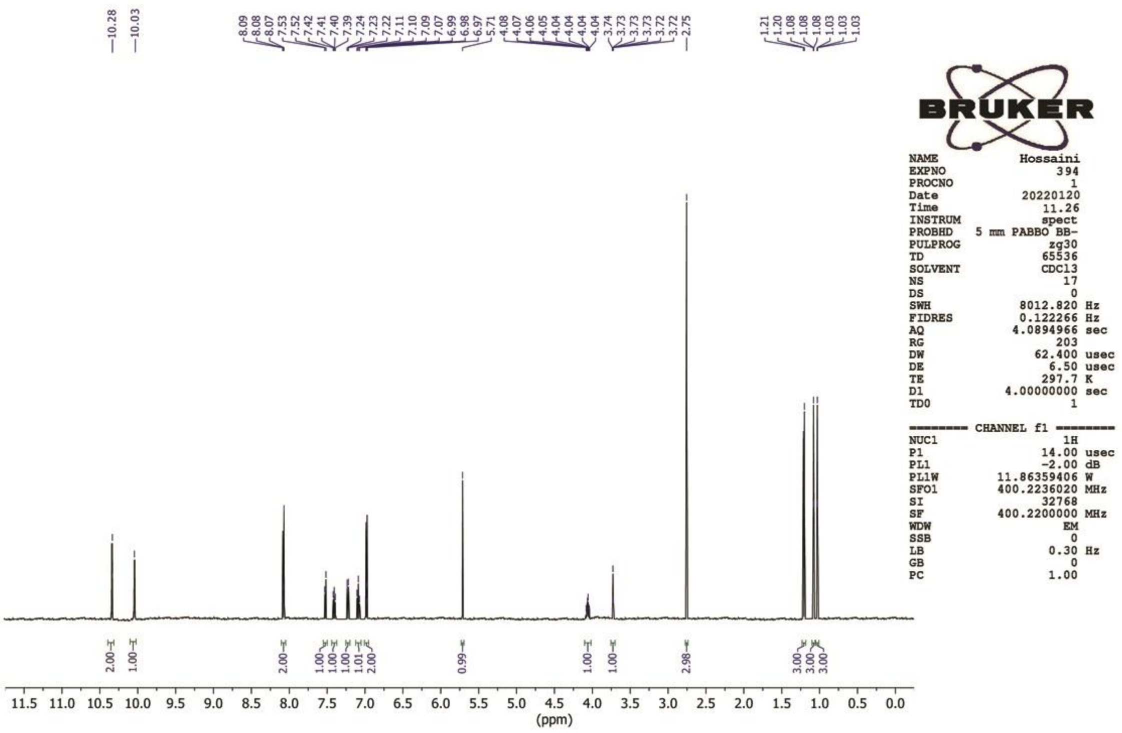

Compound 7i

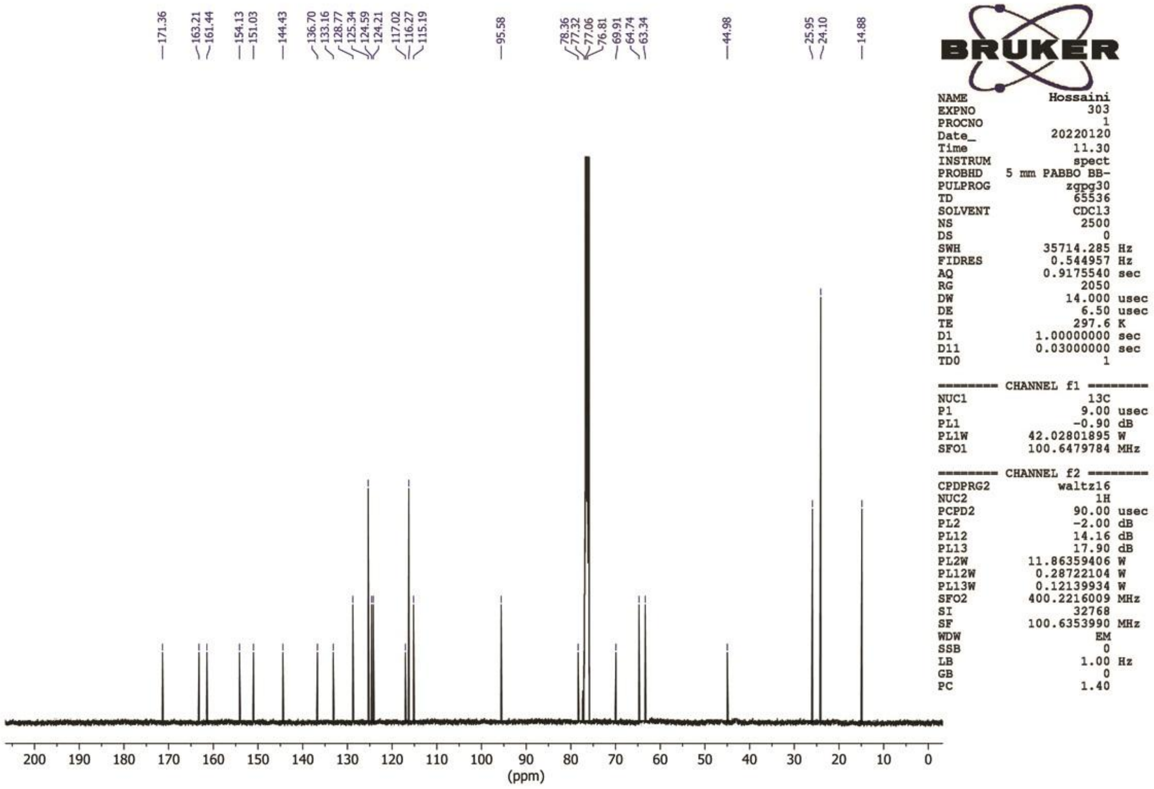

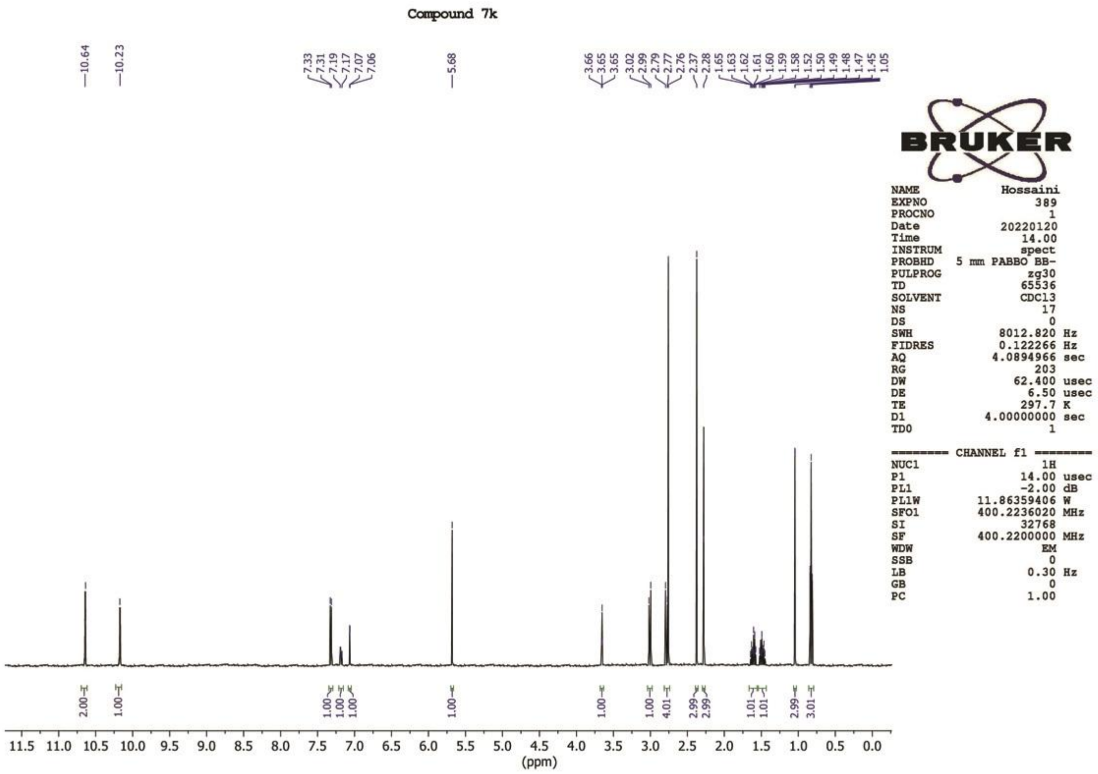
Compound 7k
(ppm)

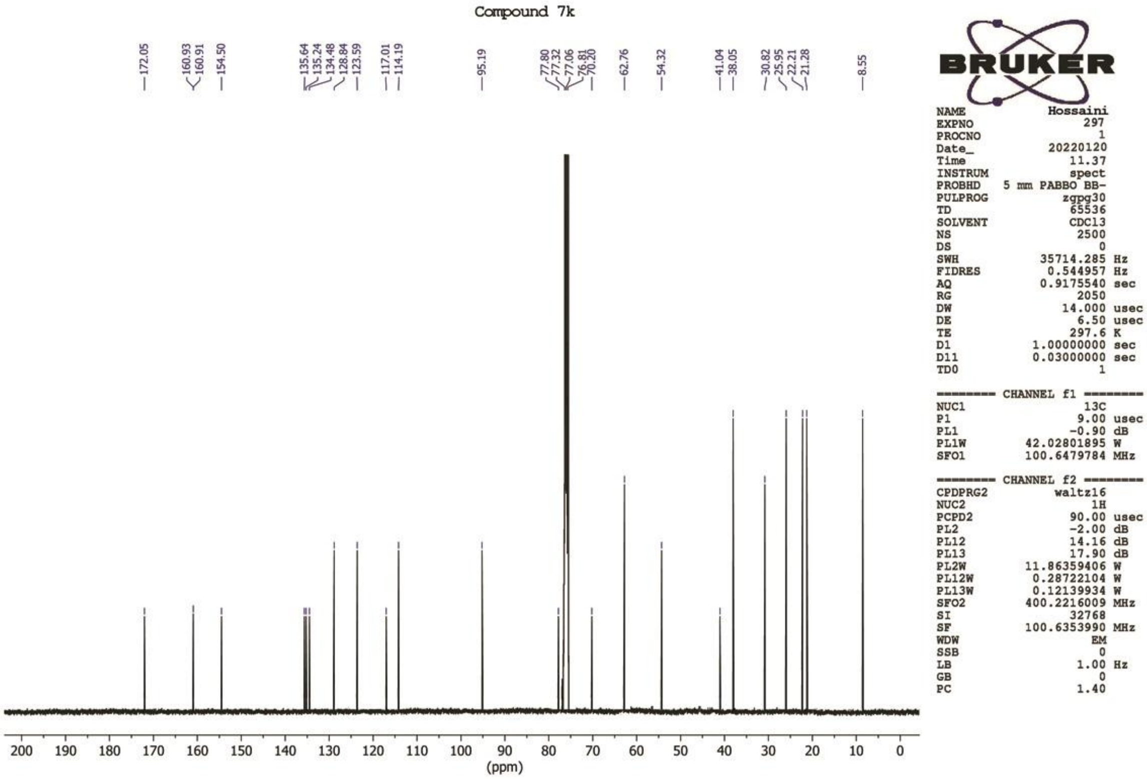
Compound 7k
(ppm)

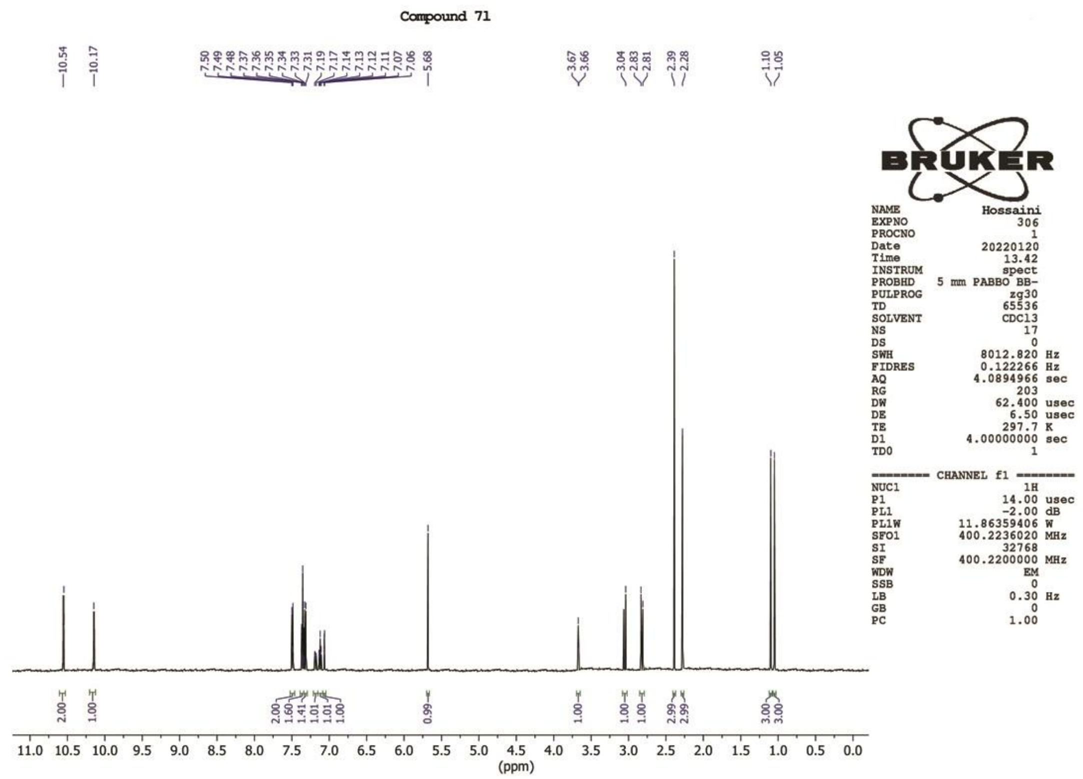
Compound 71
BRUKER
NAME Hossaini
EXPNO 306
PROCNO 1
Date 20220120
Time 13.42
INSTRUM spect
PROBHD 5 mm PABBO BB-
PULPROG zg30
TD 65536
SOLVENT CDCl3
NS 17
DS 0
SWH 8012.820 Hz
FIDRES 0.122266 Hz
AQ 4.0894966 sec
RG 203
DW 62.400 usec
DE 6.50 usec
TE 297.7 K
D1 4.00000000 sec
TD0 1
CHANNEL f1
NUC1 1H
P1 14.00 usec
PL1 -2.00 dB
PL1W 11.86359406 W
SFO1 400.2236020 MHz
SI 32768
SF 400.2200000 MHz
WDW EM
SSB 0
LB 0.30 Hz
GB 0
PC 1.00
(ppm)

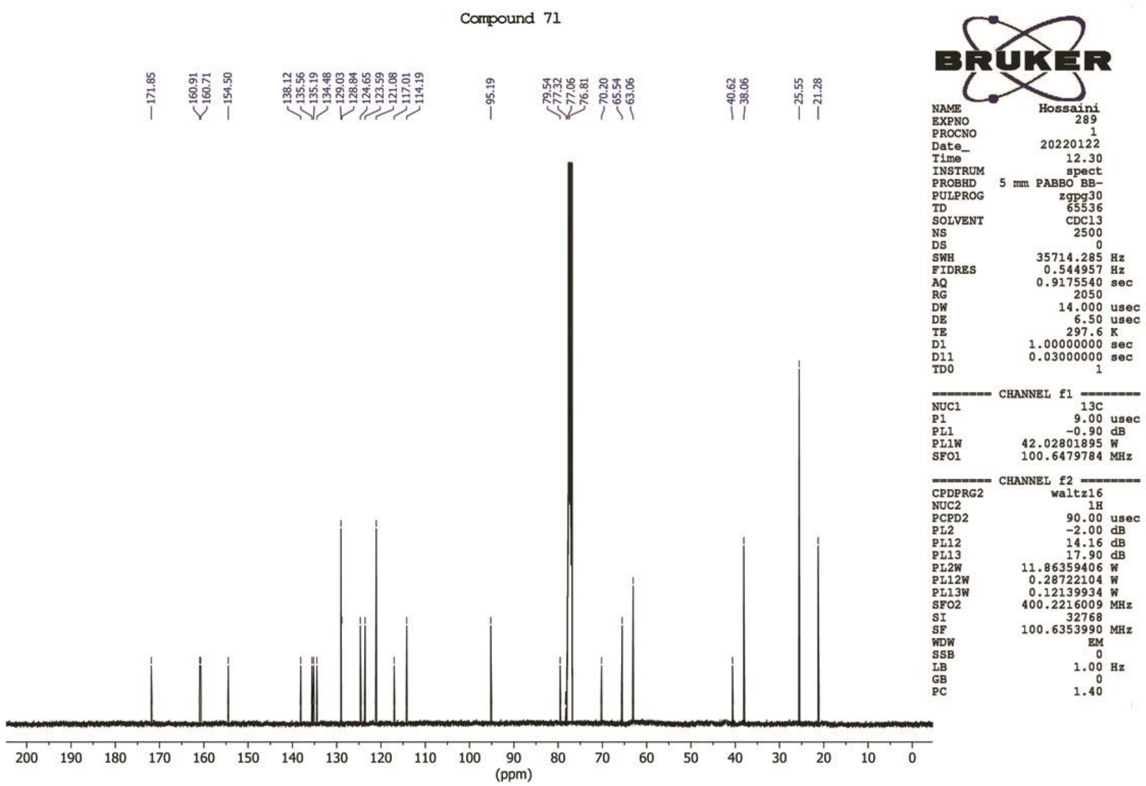
Compound 71
BRUKER
NAME Hossaini
EXPNO 289
PROCNO 1
Date_ 20220122
Time 12.30
INSTRUM spect
PROBHD 5 mm PABBO BB-
PULPROG zgpg30
TD 65536
SOLVENT CDCl3
NS 2500
DS 0
SWH 35714.285 Hz
FIDRES 0.544957 Hz
AQ 0.9175540 sec
RG 2050
DW 14.000 usec
DE 6.50 usec
TE 297.6 K
D1 1.00000000 sec
D11 0.03000000 sec
TD0 1
CHANNEL f1
NUC1 13C
P1 9.00 usec
PL1 -0.90 dB
PL1W 42.02801895 W
SFO1 100.6479784 MHz
CHANNEL f2
CPDPRG2 waltz16
NUC2 1H
PCPD2 90.00 usec
PL2 -2.00 dB
PL12 14.16 dB
PL13 17.90 dB
PL2W 11.86359406 W
PL12W 0.28722104 W
PL13W 0.12139934 W
SFO2 400.2216009 MHz
SI 32768
SF 100.6353990 MHz
WDW EM
SSB 0
LB 1.00 Hz
GB 0
PC 1.40
(ppm)

Compound 17a

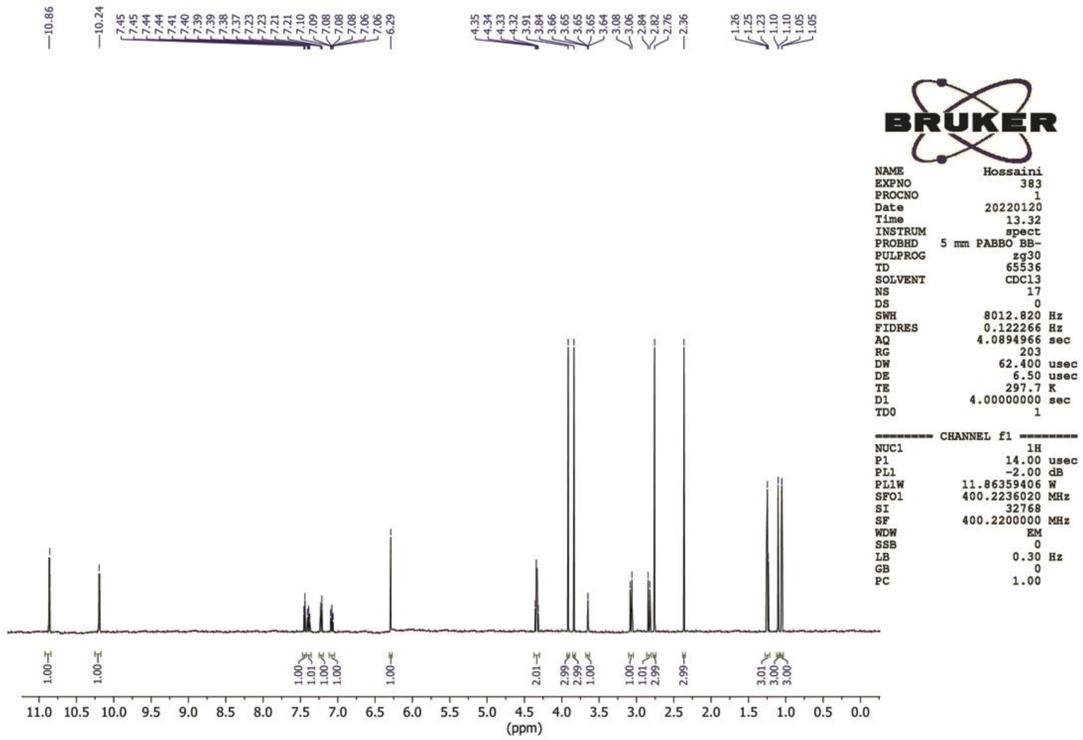

Compound 17a

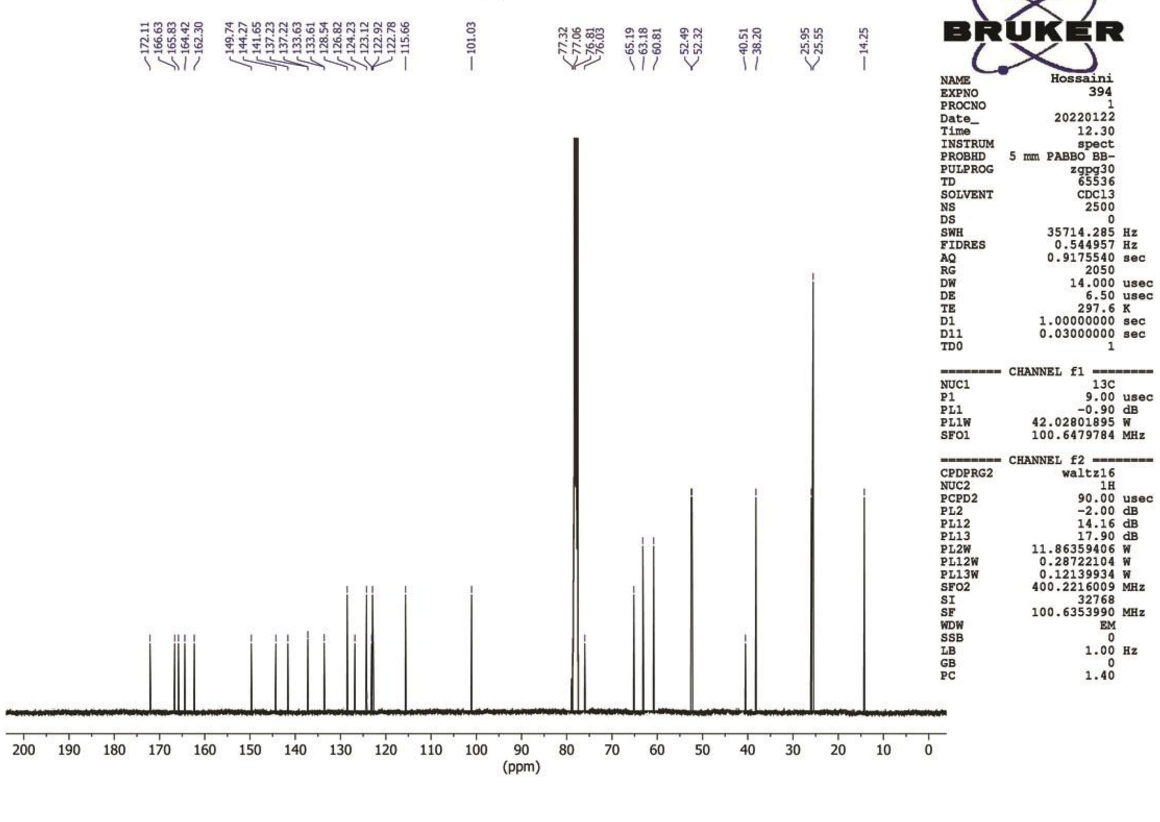

Supplement: Supplementary file 1 [file DataSheet1.PDF]
